# Sinking Our Teeth in Getting Dental Stem Cells to Clinics for Bone Regeneration

**DOI:** 10.3390/ijms22126387

**Published:** 2021-06-15

**Authors:** Sarah Hani Shoushrah, Janis Lisa Transfeld, Christian Horst Tonk, Dominik Büchner, Steffen Witzleben, Martin A. Sieber, Margit Schulze, Edda Tobiasch

**Affiliations:** Department of Natural Sciences, Bonn-Rhein-Sieg University of Applied Sciences, von-Liebig- Strasse. 20, 53359 Rheinbach, Germany; sarah.shoushrah@h-brs.de (S.H.S.); janice.transfeld@hotmail.de (J.L.T.); christian.tonk@h-brs.de (C.H.T.); dominik.buechner@h-brs.de (D.B.); steffen.witzleben@h-brs.de (S.W.); martin.sieber@h-brs.de (M.A.S.); margit.schulze@h-brs.de (M.S.)

**Keywords:** dental stem cells, iPSCs, dental stem cells immortalization, organoids, osteogenesis, angiogenesis, growth factors, low-level laser therapy, hypoxia, bone tissue engineering, scaffolds, drug release

## Abstract

Dental stem cells have been isolated from the medical waste of various dental tissues. They have been characterized by numerous markers, which are evaluated herein and differentiated into multiple cell types. They can also be used to generate cell lines and iPSCs for long-term in vitro research. Methods for utilizing these stem cells including cellular systems such as organoids or cell sheets, cell-free systems such as exosomes, and scaffold-based approaches with and without drug release concepts are reported in this review and presented with new pictures for clarification. These in vitro applications can be deployed in disease modeling and subsequent pharmaceutical research and also pave the way for tissue regeneration. The main focus herein is on the potential of dental stem cells for hard tissue regeneration, especially bone, by evaluating their potential for osteogenesis and angiogenesis, and the regulation of these two processes by growth factors and environmental stimulators. Current in vitro and in vivo publications show numerous benefits of using dental stem cells for research purposes and hard tissue regeneration. However, only a few clinical trials currently exist. The goal of this review is to pinpoint this imbalance and encourage scientists to pick up this research and proceed one step further to translation.

## 1. Introduction

Regenerative medicine is an interdisciplinary field that is concerned with finding ways to repair or replace damaged tissues and organs [1]. Stem cells are a fundamental part of regenerative medicine, and since their isolation from the bone marrow in the 1970s they have been isolated from various tissue sources [2,3,4,5]. Dental tissues such as wisdom teeth or periodontal ligaments are relatively new sources for stem cells. Dental stem cells (DSCs) are multipotent and can give rise to several differentiated cells an ability vital for tissue maintenance [6]. DSCs could be used in various areas: in basic research to study developmental processes, such as development of the tooth or disease modeling and pharmaceutical investigations, or for regenerative medicine, especially if induced pluripotent stem cells (iPSCs) are generated from them. This review introduces the isolation, characterization, and subsequent applications of DSCs in research. These include the generation of immortalized dental stem cell lines, iPSCs, and organoids. Osteogenesis and angiogenesis are vital processes for hard tissue regeneration; the potential of DSCs for these two processes will be discussed in this review as well. Another focus will be the effect of growth factors and environmental modulators on the differentiation of DSCs. The potential of translating the use of DSCs for bone tissue regeneration will be the focus in this review. In detail, multiple strategies for utilizing DSCs in hard tissue regeneration such as cell sheets and cell free therapy will be explored. In addition, scaffold-based approaches, including the different scaffold materials and drug release strategies that could be used to support the use of DSCs in tissue regeneration will be discussed. Finally, current clinical trials using dental stem cells will be summarized.

## 2. Stem Cells Isolated from Dental Tissues

Adult stem cells (ASCs) can be found within most of the tissues of the body. Their main functions are to develop, regenerate, and maintain the steady state of the respective tissues [6]. Stem cells achieve this by two main characteristics: they can differentiate into mature cell types with a specialized function and by keeping their feature of self-renewal [7,8,9]. Stem cells can differentiate into an intermediate state, which is a progenitor state defined by a reduced potency before they reach the fully differentiated state. Thus, ASCs found in tissues are already partly committed toward certain cell types. The lineage-specific differentiation capacity of tissue-specific adult stem cells is still multipotent. This ability to differentiate is based on the organ or tissue of origin [9,10]. Precursor cells have been found so far in a variety of organs such as the brain [11], kidney [12], and lungs [13], while multipotent stem cells were found in bone marrow [2], peripheral blood [14], adipose tissue [15], and in the dental tissues [16,17,18].

**Figure 1:**
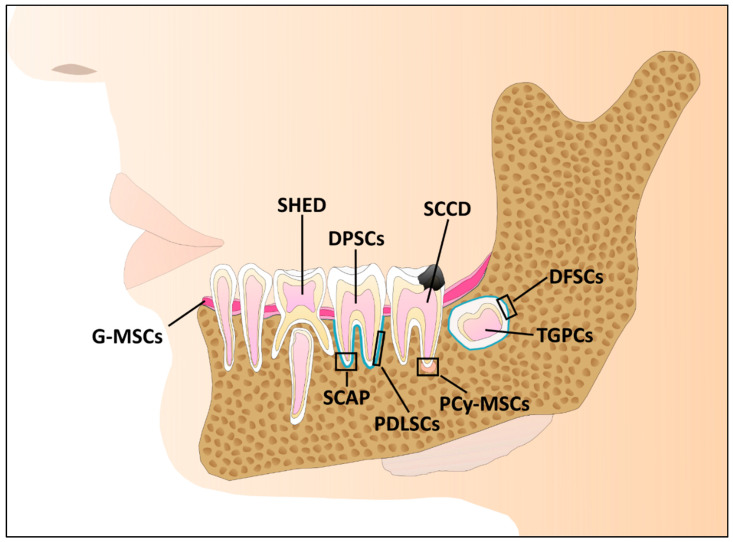
Overview of the stem cells with an oral and dental origin. The dental tissue is a rich source of multipotent and mesenchymal stem cells. Dental pulp stem cells (DPSCs), dental follicle stem cells (DFSCs), stem cells from exfoliated deciduous teeth (SHED), stem cells from the apical papilla (SCAP), periodontal ligament stem cells (PDLSCs), gingival mesenchymal stem cell (G-MSCs), tooth germ stem cells (TGSCs), mesenchymal stem cells from periapical cysts (PCy-MSCs), and dental pulp stem cells from carious deciduous teeth (SCCD) can be isolated from this region.

Dental development starts preceding a child’s birth and continues beyond until the permanent teeth replace the primary, deciduous teeth. As a result of that, DSCs can be obtained from immature, developing tissue as well as from mature, developed tissues, whereas for the immature teeth, the dental follicles have a higher stemness and thus a higher potency [19]. Therefore, there is a variety of stem cells isolated from dental tissues, and one feature they share is their extraordinary plasticity and differentiation potential (Figure 1) [20]. These stem cells include the following:

1. Dental pulp stem cells (DPSCs), which can be isolated from dental pulp tissue (mature teeth) [21];
2. Stem cells isolated from exfoliated deciduous teeth (SHED) [22];
3. Dental follicle stem cells (DFSCs), which can be isolated from wisdom teeth that have not crossed through the gum [23];
4. Tooth stem cells (TGSCs) isolated from the tooth germ [24];
5. Gingival mesenchymal stem cells (G-MSCs) isolated from gingival tissue [25];
6. Stem cells isolated from the apical papilla (SCAP) [26,27];
7. Periodontal ligament stem cells, which as their name implies are isolated from the periodontal ligaments surrounding the teeth (PDLSCs) [28].

Additionally, DSCs can be isolated from diseased dental tissues such as cysts or carious teeth. Periapical cysts that result from infection of the tooth apex and the adjacent bone contains periapical cyst-MSCs (PCy-MSCs) [29,30]. Dental pulp stem cells from carious deciduous teeth (SCCD) and carious permanent teeth (CDPSCs) can be collected when the infected tooth is surgically removed [31,32] (Figure 1). Most of the sources are usually medical waste, making them easily accessible. They can be isolated with simple techniques compared to stem cells of other origins where harvest procedures are more invasive [33].

### 2.1. Isolation and Characterization of Stem Cells from Dental Tissues

Dental pulp stem cells have been isolated in vitro for the first time in 2000 by Gronthos and colleagues [21]. The cells were collected from the dental pulp, which is the inner part of teeth harboring a loose connective tissue [34]. DPSCs were described as mesenchymal stem cells (MSCs) since their phenotypical structure was similar to that of mesenchymal stem cells from the bone marrow [35]. In general, there are two major techniques to isolate stem cells from teeth. Either the teeth can be digested with enzymes, or pieces of the tooth are put in culture dishes for outgrowth of the cells. Later, the cells can be sorted and isolated by the detection of specific markers. The DPSCs were isolated from permanent teeth by digestion. Collagenase type I for the generation of single-cell suspensions was used followed by clonal expansion [36]. An advantage of DPSCs compared to bone marrow MSCs (BM-MSCs) that Gronthos and colleagues described was the higher yield of MSCs from the dental region [21]. Miura and colleagues isolated SHED for the first time using the enzymatic technique enzymatic, and since, then both isolation techniques have been used [22,37]. Dental follicle stem cells can be isolated by an enzymatic digestion with collagenase I and trypsin after separating the dental follicle tissue from the dental crown. Afterwards, the cells can be seeded, and only the DFSCs will adhere to plastic cell culture dishes [38]. The isolation of SCAP from the apical papilla can be done accordingly [27]. The first report of the successful isolation of PDLSCs was published in 2004 by Seo and colleagues [28]. In line with previous described isolation methods, an enzymatic digestion with collagenase I was done to isolate them. In contrast to that, G-MSCs were first successfully isolated by Zhang and co-workers in 2009, and since then, there have been several variations for the isolation and culture protocols reported [39,40]. Tooth germ stem cells were first isolated by Ikeda and colleagues in 2008 [24]. The tooth germ tissue was separated and mechanically shredded with a scalpel and then placed in plastic cell culture dishes with growth medium. The TGSCs grew out of the tooth germ tissues after an incubation for a week [41]. The isolation of PCy-MSCs from periapical cysts, obtained from surgery, starts with a mechanically disruption of the cystic wall. Afterwards, the cystic tissue is shredded and subjected to enzymatic digestion with collagenase I. The shredded material can be placed in cell culture dishes to allow the outgrowth [42]. The isolation of SCCD was done by Werle and colleagues and was done accordingly [31].

**Figure 2:**
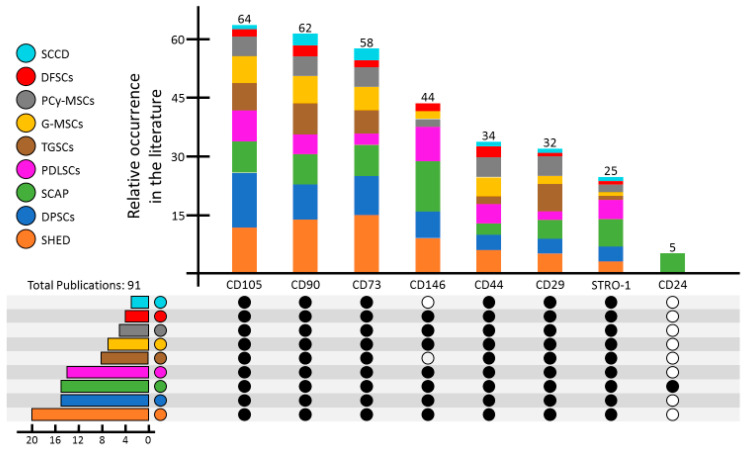
Marker expression profile of the different stem cells isolated from dental tissues. For this analysis, 91 publications were analyzed. The publications were chosen by their content focusing on each dental stem cell type and their isolation and characterization. We analyzed 20 SHED publications, 15 DPCs and SCAP publications, 14 PDLSCs publications, 7 G-MSCs publications, 4 DFSCs publications, and 3 SCCD publications. The black circles indicate that the marker was described to be expressed in the literature, while the white circles indicate that the expression of the marker has not been described in the literature yet.

The Mesenchymal and Tissue Stem Cell Committee of the International Society for Cellular Therapy set minimal criteria to define stem cells as MSCs [43,44,45]. Among the defined criteria, the CD markers CD105, CD90, and CD73 are supposed to be positive, while CD11b, CD14, CD34, and CD45 should be negative. DSCs share the described common features with ASCs isolated from other sources [46]. The marker expression of all these different mesenchymal stem cells from the oral origin is in general similar, but it also shows some small differences. Figure 2 summarizes the detailed marker expression profile of the different DSCs. The analysis of the most common markers was done by a literature search of 91 total publications published within 2000 and 2020 (Figure 2) [22,24,27,30,47,48,49,50,51,52,53,54,55,56,57,58,59,60,61,62,63,64,65,66,67,68,69,70,71,72,73,74,75,76,77,78,79,80,81,82,83,84,85,86,87,88,89,90,91,92,93,94,95,96,97,98,99,100,101,102,103,104,105,106,107,108,109,110,111,112,113,114,115,116,117,118,119,120,121,122,123,124,125,126,127]. The literature was scanned for articles discussing the different stem cell types and their isolation and characterization. Since some stem cells were discovered earlier than others, different amounts of articles were found and used to describe the marker expression of these stem cell types. The most common positive markers expressed in all multipotent stem cell types from dental tissues are CD105, CD90, CD73, and CD146, whereas CD146 was not described to be expressed in SCCD yet. This might be due to the low number of publications for that cell type [31,128,129].

To investigate the most common expression pattern of positive markers in SHED, 20 publications were analyzed and summarized in Figure 2 [22,47,48,49,50,51,52,53,54,55,56,57,58,59,60,61,62,63,64,65]. In 15 of them, CD73 was described to be expressed; the other expressed positive markers of SHED were CD90 (14) and CD105 (12). The same three positive markers were also described to be expressed on DPSCs. From 15 publications analyzed, CD105 was described to be expressed on SHED in 14 publications, while CD73 and CD90 were described to be expressed in 9 and 10 publications [58,66,67,68,69,70,71,72,73,74,75,76,77,78,79]. The analysis of marker expression on SCAP was as follows: 15 publications were analyzed, and the most described markers expressed were CD146 (13), CD73 (8), CD90 (8), and CD105 (8) [27,66,80,81,82,83,84,85,86,87,88,89,90,91,92]. SCAP is the dental stem cell type where CD146 is described as the most prominent positive marker. Another special marker in this this dental stem cell type is CD24, which was described in comparison with all other dental stem cell types as an exclusive marker for these cells. A similar pattern of positive markers expressed was documented for PDLSCs. Fourteen publications were analyzed whereby nine of those mentioned CD146 as a positive marker, eight mentioned CD105, and five mentioned CD90, STRO-1, and CD44. CD73 was only documented as a positive marker for PDLSCs in three of the 14 analyzed publications [67,93,94,95,96,97,98,99,100,101,102,103,104,105]. In TGSCs, a total of eight publications were analyzed, and CD90 was mentioned in all of them [24,106,107,108,109,110,111,112]. CD105 as well as CD29 were mentioned in seven of those eight publications as a positive marker for TGSCs, while CD73 was mentioned in six publications. For the positive marker expression pattern of G-MSCs, seven publications were analyzed [113,114,115,116,117,118,119], all of them describing CD90 and CD105. CD73 was mentioned in six publications, and in five of the seven publications, CD44 was described. Just five publications were found that discuss markers in PCy-MSCs [30,120,121,122,123]. In all these publications, the same set of markers CD29, CD44, CD73, CD90, and CD105 were found to be expressed. DFSCs markers were described in four publications [124,125,126,127]. In three of those publications CD44 and CD90 were found, and in two of those publications CD73, CD105, and CD146 were found. Taken together, from 91 publications analyzed, CD105 was depicted in 64 publications, CD90 was depicted in 62 publications, and CD73 was depicted in 58 publications to characterize the multipotent dental stem cells. CD146 was not described to be found yet on SCCD and on TGSCs, and CD24 seems to be a positive marker exclusively expressed on SCAP.

Although there is a wide variety of dental stem cells, they all display the required at least positive markers to be defined as mesenchymal stem cells. However, working with primary cells is time consuming and difficult. Therefore, immortalized cell lines are favorable for most scientific questions, which can be addressed. This will be discussed in the next section.

### 2.2. Dental Stem Cells-Derived Cell Lines

Dental stem cells, similar to most ASCs, are subject to the Hayflick limit, which is the number of times cells can divide until cell division ceases, meaning that the cells cannot be cultured indefinitely due to cell senescence [130]. This phenomenon hinders research efforts; thus, establishing immortalized cell lines of MSCs isolated from different body regions would make stem cell research more standardized and is therefore highly desirable [131]. Immortalized cell lines have been established from different kinds of human and mouse dental stem cells, with most researchers focusing on immortalizing DPSCs [132,133,134], including SHED [130,135], while less work has been done on dental epithelial stem cells (HERS/ERM) so far [136,137,138]. Most often, viral oncogenes (e.g., simian vacuolating virus 40 (SV40) large T-antigen or E6/E7) are used to immortalize the cells of interest [139]. SV40 immortalization is effective in most cell types; it acts on the tumor suppressors p53 and Rb, while it also promotes the expression of the human telomerase reverse transcriptase (hTERT) to overcome telomer shortening [132,139]. However, viral oncogene immortalization is often associated with chromosome abnormalities, polyploidies, and changes in gene expression [132,139]. The vectors for transducing the foreign gene include using lentiviruses. However, they have the drawback of random integration into the genome with the risk of unwanted side effects of upregulation of oncogenes or suppression of the tumor suppressor gene. Liposome delivery is one alternative to express viral oncogenes, which was shown to maintain osteogenic differentiation capacity and gene expression in the produced cell lines, but episomal vectors are stable for only a short time and will be lost afterwards [140]. There are ongoing efforts to find other possibilities to create cell lines, which better represent the primary cells from which they are derived.

The immortalization of different dental cells was shown in several studies by transduction with hTERT alone [130,133,135,141,142]. Cell immortalization using hTERT overexpression controls senescence caused by the shortening of telomers by adding telomeric DNA repeats at the end of cell division [133]. In most studies immortalizing dental stem cells, hTERT is ectopically expressed using lentiviral vectors [135]. However, the outcome of studies applying hTERT overexpression for immortalizing dental stem cells remains controversial. In the study by Egbuniwe and colleagues, increased survival of DPSCs with immortalization using the hTERT system is reported, while gene and MSC marker expression was similar and differentiation capacity was even stronger than found in the parental cells. Only CD166 and CD146 were reduced after long passaging [133]. Urraca and colleagues reported that the osteogenic potential in immortalized DPSCs was decreased, but the neuronal differentiation from these immortalized DPSCs appeared very similar to the non-immortalized controls. On the other hand, their expression pattern was distinct from non-immortalized DPSCs, which they suggest might be due to hTERT repressing certain transcripts [142,143]. In SHED immortalized with stable hTERT expression, the proliferation rate and differentiation capabilities were retained, while the expression of STRO-1, CD146, Oct4, Nanog, and CD34 was decreased, and no tumorigenicity or genetic instability was reported [135]. In other reports, it was shown that hTERT-immortalized SHED maintained differentiation capacity toward the neural linage, and no in vivo tumor formation was found, but abnormalities in soft agar colony formation and slight genetic instabilities were more pronounced [141]. In a more recent study, immortalized DPSCs were produced using an R24C mutant of cyclin-dependent kinase 4, cyclin D1, and TERT (K4DT-immortalization), since they reported that TERT alone was not sufficient for immortalization [132]. In this study, a stable chromosome pattern was shown, with an absence of senescence and a higher proliferation rate and differentiation capability in comparison to the primary cells in the investigated linage [132]. Recently, the immortalization of SHED, pre-chosen cells with favorable stem cell characteristics such as octamer-binding transcription factors 3/4 (Oct3/4) expression and good reprogramming efficacy, was also reported using both HPV16-E7 and hTERT inserted by piggyBac transposon-based delivery [130]. The authors describe the immortalized cells from both methods as indistinguishable from the primary cells in terms of proliferation, stemness gene expression, morphology, and differentiation capacity, while also maintaining anchorage-dependence and non-tumorigenicity, which are usually drawbacks of immortalization, proposing a promising non-lentiviral approach [130].

An example of the application of immortalized DSCs is the investigation of the underlying mechanisms of odontogenesis and osteogenesis. Wu and colleagues showed the immortalization of SCAP from mice with floxed bone morphogenetic protein 2 (*Bmp2*) genotype, meaning it carries LoxP sites upstream and downstream of the *bmp2* gene. The cells were isolated from molars of mice and used to study the role of bone morphogenetic protein 2 (BMP2) in knockout cells during tooth and bone development, omitting systemic effects. They are also valuable because the BMP2-KO mice are infertile [144]. Furthermore, the same group also studied the role of BMP2 knockout cells, which were transduced with Ad-Cre-GFP to excise the *bmp2* allele, and they found reduced cell growth, G2-cell cycle arrest, and a reduction of bone associated proteins, indicating an important role of BMP2 for cell growth, cell cycle, and osteogenesis [145]. Another interesting gene in this context is *Fam20c*, which encodes a kinase that phosphorylates several secretory proteins, including small integrin-binding ligand N-linked glycoproteins (including bone sialoprotein (BSP), osteopontin (OPN), dentin matrix acidic phosphoprotein 1 (DMP1), dentin sialophosphoprotein (DSPP), matrix extracellular phosphoglycoprotein (MEPE)) and fibroblast growth factor 23 (FGF-23) [146]. Its exact role in osteogenesis/dentinogenesis is still under investigation. In this context, immortalized SCAP cell lines from Fam20c floxed mice have been proven valuable [146]. In cells where Fam20c was abrogated, the proliferation, migration, and mineralization were lowered and the BMP signaling was attenuated, indicating a possible interplay giving room for further research [147]. In another study, SCAP from mouse incisors were shown to be induced to undergo osteogenesis/odontogenesis using BMP9 [148]. They further investigated the role of Wnt and β-catenin signaling in this context and found that the silencing of β-catenin weakens the BMP9-mediated effect, while Wnt3A acts in a synergic manner [149]. Furthermore, mouse incisor SCAP have also been immortalized using SV40-T antigen flanked with Cre/LoxP sites, yielding a reversible immortalized cell line, while retaining its multi-lineage differentiation capabilities [148].

Epithelial stem cells, which give rise to ameloblasts and are crucial for enamel formation, are found sparsely and in a few locations only [138]. Hertwig’s epithelial root sheath/epithelial rests of Malassez (HERS/ERM) are necessary to study epithelial–mesenchymal interactions, which is necessary for tooth root formation and guiding odontogenic differentiations. They are also interesting for studying epithelial–mesenchymal transition, which is required for cementoblast differentiation [136,137]. HERS can be isolated in a quiescent state from the periodontium in humans, which is quite small, thus providing only a few cells. Nam and colleagues reported the immortalization of HERS/ERM cells from the human periodontium with SV40 LT [138]. The resulting cell line retains characteristics of the primary cells. The cell line could be expanded for over 20 passages and seems to undergo epithelial–mesenchymal transition induced by transforming group factors-beta 1 (TGF-β1), indicating similar behavior to primary HERS [138]. A limitation of these human cells isolated from ERM is that the cells seem to be terminal products of HERS and thus not resemble HERS during development [137]. For this reason, Li and colleagues used HERS from unerupted molars of mice for the immortalization of two cell lines. They described heterogenicity in their immortalized cell lines: both could undergo epithelial–mesenchymal transition to adapt a cementoblast phenotype, but only one could produce cementum-like structures, while the other was capable of inducing odontogenic differentiation in DSCs. This is indicating the presence of subpopulations of HERS [137]. They also identified differences in the expression of molecules involved in extracellular matrix receptor interaction, which is proposed for influencing the phenotype and function of cells, hence suggesting an explanation for the heterogenicity of the cell lines [137]. Using one of these cell lines, they could identify that Smad and BMP signaling are crucial for triggering the odontogenic differentiation of DPCs in vitro [136].

Immortalized DSCs cell lines provide a useful tool for research in studying cellular and developmental processes. The creation of an immortalized cell line from primary cells is especially useful when applied to scarce stem cell types such as epithelial stem cells. Another, still novel type of cell line is described in the next subsection: the production of induced pluripotent stem cells from dental stem cells. Induced pluripotent stem cells lack the typical features of the targeted cells, but gain the pluripotent state and thus can be applied for other applications as immortalized but still differentiated cell lines. However, the mixed epigenetic pattern composed of the target and the goal cell always should be considered while working with iPSCs.

### 2.3. Production of iPSCs from Dental Stem Cells

One of the limitations of adult stem cells is their limited differentiation potential and decreasing proliferation in long-term culture [150]. Immortalizing the stem cells is one option to bypass this problem, but immortalization has its downsides as well. On the other hand, pluripotent stem cells such as embryonic stem cells (ESCs) and induced pluripotent stem cells (iPSCs) have higher potential and are suggested to be unlimitedly expandable in vitro [151]. iPSCs were developed in 2006 by Yamanaka and Takahashi, who discovered that using the transcription factors Oct3/4, Sox2, Klf4, and c-MYC made it possible to reprogram somatic cells into an ESC-like state [152,153]. The group of Thomson showed that this was also possible with the factors Oct4, Sox2, Lin28, and Nanog [154]. iPSCs are commonly reprogrammed from somatic cells, but it has been published that the cell origin affects the efficiency of reprogramming and the amount of transcription factors needed. Examples of that are neural stem cells, which were shown to be reprogrammed by using Oct4 only [155] or keratinocytes, which were about 100× more efficiently reprogrammed compared to fibroblasts [156]. Due to their stemness, strong proliferation, and endogenous expression of several pluripotency markers, DSCs are considered to be more efficiently reprogrammed [157]. Additionally, wisdom teeth or lost milk teetare medical waste. Hence, they are easy to obtain and less prone to have damaged DNA resulting from exposure to external environmental factors, such as sunlight, which can be a drawback of fibroblasts [157]. DPSCs also showed high proliferative activity and are a good accessible source, making them interesting candidates for iPSC generation [158,159].

The reprogramming of dental stem cells seems promising. DPSCs and SHED, SCAP, dental pulp cells [160], cells extracted from wisdom tooth [158], periodontal, gingival, and mucosal fibroblasts [161,162] have all been successfully reprogrammed to iPSCs using different techniques. So far, multiple reprograming methods have been reported for generating iPSCs from DSCs. Generally, the methods feature either an integrative or a non-integrative delivery of the reprogramming factors [158]. Most commonly, lentiviral vectors are used due to their high efficacy. Meanwhile, these vectors display the drawback of random integration into the genome and often continuous expression of the transgene, displaying a great safety issue that gets in the way of considering their clinical application [159,163]. The use of non-integrative reprogramming methods is reducing these risks; however, one drawback is their lower reprogramming efficacies [159]. One approach to overcome this is a careful selection of cell sources that can make non-integrative techniques more feasible [158]. Zou and colleagues described an innovative transgene-free method to reprogram SCAP cells with a lentiviral “stem cell cassette”, which contains LoxP sites and can be excised via a plasmid delivering Cre after reprogramming is completed, so that only the viral LTR remain integrated [164]. In addition, non-integrative Sendai viral delivery has been shown to work successfully in reprogramming natal DPSCs from teeth present at birth [165] and mature adult DPSCs [166,167] with high efficacy [165]. The use of plasmid vectors for reprogramming DPSCs have been shown in several studies [168,169], while reports about their reprogramming efficacy remains inconsistent. DPSCs were reprogrammed without using c-MYC, which is a gene that is problematic due to its possible oncogenic effects [170]. The generated iPSCs maintained iPSC characteristics as proven by their ability to differentiate toward the neural lineage, paving another step toward safer iPSC technologies [170]. More recently, episomal plasmid reprogramming was shown to be feasible using xeno-free culture conditions and feeder-free culture by applying an inhibitor cocktail (SMC4 cocktail), thus adapting it to a clinical approach at the same time. The authors showed fast reprogramming with similar efficacies as compared to retroviral reprogramming, leading to high cell viability, survival, and high clonality of iPSC [169].

ESC and iPSC were originally cultured always on feeder-layer cells [153]; Saitoh and colleagues reported a benefit of the mouse embryonic fibroblast (MEF) feeder layer during reprogramming over (SIM)-derived 6-thioguanine- and ouabain-resistant (STO) feeder layer cells on DPCs, possibly by produced growth factors of the feeder cells [168]. Chen and colleagues reported that DPC-iPSC could be grown also on DPC-derived ECM retaining the undifferentiated state, instead of a feeder-layer dependent culture. Hypoxic conditions during the early stage of reprogramming could improve the efficacy of the reprogramming process and change the gene expression pattern in the DPCs, indicating a complex mechanism behind this. Meanwhile, hypoxia during later stages of the reprogramming reduced the efficacy [171]. Overall, the improvements of culture conditions toward chemically defined substances and feeder-free culture are crucial for making stem cell research more reproducible. Its use together with a refined reprogramming, preferably using episomal vectors due to safety concerns, will be an important step toward clinical applicability.

Differences in iPSC generation from cells with different maturities and tissue locations have been described in several studies. It was described that SHED are more immature in comparison to DPSCs isolated from older teeth and are more easily reprogrammed [157,172]. Beltrão-Braga and colleagues pointed out that the immature DPSCs do express markers of both MSCs and ESCs (e.g., SSEA & Oct3/4), but generally share characteristics with the latter [157]. The methylation pattern of pluripotency markers in DPSCs was found to be closer to that of ESCs, possibly lowering the reprogramming barrier in these cells [169]. With this in mind, iPSCs derived from DPCs that were isolated from different developmental stages (crown completed (CC), root forming (RF) and root completed (RC)) of the wisdom teeth also showed different efficacies in reprogramming, with those at more immature stages (CC and RF stage) being more readily reprogrammed [173]. A link of Distal-Less Homeobox 4 (DLX4) expression with the readiness for iPSC generation was suggested since dental pulp cells show high expression; DLX4 is generally found in conditions of abnormal tooth development as well. Others describe alkaline phosphatase (ALP) and OCT3/4 expression as characteristic for the reprogramming efficacy [174]. Recently, the expression of paired box gene 9 (PAX9) and human endogenous retrovirus (HERV-FRD) was also described to affect the reprogramming efficacy in DPSCs, as shown by improved efficacy when overexpressing PAX9 and knocking down HERV-FRD [169]. Additional support for the role of immaturity affecting the reprogramming efficacy is found in the studies by Toriumi and colleagues and Honda and colleagues, who reported that stem cells from the radicular pulp do express higher levels of Klf4 and proliferate more strongly. In addition, they are more readily reprogrammed than coronal pulp stem cells, which they base on their different stages in development [70,175]. In line with the previous reports, Soda and colleagues proposed that a higher content of immature stem cells yields better reprogramming. They further investigated the downregulation of mesenchymal markers and upregulation of pluripotency markers during reprogramming, coming to the conclusion that intermediate stages during reprogramming must exist [172]. From the six dental cell populations they tried to reprogram only one gained iPSC characteristics after the first transfection while the other cell populations maintained their parental morphology. Repeated transfections later also yielded an iPSC-like state for these cells. However, interestingly, already after the first transfection, the cells changed to intermediate differentiation stages characterized by altered molecular markers and higher degree of multipotency [172]. They also found that these intermediate cells do not cause teratoma formation in mice while also having increased differentiation capacities [172]. This suggests that not necessarily a complete reprogramming to an ESCs-like state may be required based on which application is desired, which reduces the risks typically found in iPSCs. Inada and colleagues showed that iPSC derived from SHED could be reprogrammed further to a more naïve state via a cocktail of 2i-kenpaullone and forskolin. This was confirmed by the increased expression of REX-1 and SSEA-1, decreased expression of FGF-5, smaller nuclei, and stronger proliferation. Thus, for studying developmental processes, these naive iPSC may be more favorable [176].

Dental tissue-derived iPSCs were differentiated into hepatocyte-like cells [177,178], retinal pigment epithelial-like cells [179,180], neuron-like cells [142,181,182], mesenchymal stem cell-like cells [183,184], neural crest-like cells [185], dental epithelial stem cell-like cells [186], and osteoblast-like cells [183,184]. However, the application of iPSCs in the clinics is strongly limited especially due to their capability for teratoma formation; hence, a predifferentiation to multipotent stem cells is often preferred when considering possible in vivo or clinical applications. Commonly, iPSC are differentiated toward induced mesenchymal stem cells (iMSCs), which are multipotent and thus are able to differentiate into the osteogenic, chondrogenic, and adipogenic lineage [187]. In comparison to iPSCs derived from fibroblasts, SHED-derived iMSCs and also PDL-derived iMSCs show a higher osteogenic potential, presumably by retaining a part of the epigenetic memory of their parental cells [183,184]. Regrettably, induced pluripotent stem cells were found to acquire mitochondrial DNA (mtDNA) mutations, for which the cells must be screened before further application. Meanwhile, the differentiation toward iMSCs does not seem to induce further mtDNA mutations but is reducing the teratoma formation significantly [151]. iPSC derived from DPSCs showed a strong tendency to differentiate toward neural linages, which is also considered to be a partly retained memory of the original cell type, because these are cells of ectomesenchymal origin [142,181,185]. As for other tissues, which are currently still hard to reprogram, the left behind epigenetic signature and expression profile could prove advantageous in differentiating exactly into these parental cell types, progenitors or lineages derived from them [188].

The iPSCs derived from dental tissues also have a strong osteogenic potential. Osteogenic differentiation from iPSCs is often done after a predifferentiation toward the mesenchymal lineage. It was reported that iMSCs from PDL-iPSCs and SHED-iPSCs could more efficiently undergo osteogenesis than iMSCs derived from lung or gingival fibroblast iPSCs [183,184]. iPSCs have also been generated from DPCs of patients with cleidocranial dysplasia, which is a genetic disease characterized by a loss of function of Runt-related transcription factor 2 (RUNX2). This iPSC line could be used to study the effects of RUNX2 expression in processes of this disease. The most pronounced alteration described in this study was a hypertrophy of chondrocytes in teratoma formed by these iPSCs. The process of osteogenesis in these cells remains to be investigated but is of interest because RUNX2 is a key player in osteogenesis [167]. Recently, it was shown that epithelial stem cell like cells can be generated from SHED-iPSCs. Dental epithelial stem cells are sparsely found in the adult as HERS/ERM cells disappear when the teeth erupt. However, the epithelial–mesenchymal interaction is crucial for tooth development. Hence, most studies focus on mouse incisors, where dental epithelial stem cells are still accessible [186].

Tooth engineering and organoid formation is strongly limited to the population of epithelial tissue able to give rise to ameloblasts and successfully providing epithelial–mesenchymal interactions required for tooth formation in humans. Until now, research has been mainly focused on cells obtained from rodents or pig embryos. iPSC-derived dental epithelial cells, together with iPSC-derived neural crest cells, could provide a powerful tool for tooth engineering. Moreover, DSCs-derived iPSCs could also be used for disease modeling and investigating the pathogenesis of these diseases. One possibility to do so is the generation of organoids. They can also be produced from dental stem cells itself depending which scientific questions will be investigated.

### 2.4. Organoids from Dental Stem Cells

Cell culture in three dimensions gained increased attention over the years. It has a closer resemblance to physiological processes, which makes it interesting, as 3D culture provides a favorable microenvironment that 2D culture cannot [189]. Small animal models are not always able to mimic human diseases or development. Since data obtained from 2D cultured cells are only limitedly transferable to the in vivo situation, 3D settings are developed to more closely mimic the respective tissue [190]. Three-dimensional (3D) culture systems without scaffolds include spheroids, aggregate formation, but also organoids and organ germs [191,192].

Organoids and organ germ are both 3D structures resembling organs more closely. Both were tremendous steps toward modeling of tissue development and tissue regeneration. In embryogenesis, organ germs give rise to organs and are often the result of reciprocal interactions of mesenchyme and epithelium, which are already fate determined by so-called organ-forming fields [192]. The first report of a dental “organ germ” was in 2007, where epithelial and mesenchymal cells were isolated from an embryonic tooth germ. By the compartmentalization of mesenchymal and epithelial cells in high density, Nakao and colleagues were able to replicate the induction of tooth germs. In subsequent publications, they showed that it was possible to generate also other functional ectodermal organs [192,193,194,195,196,197,198]. They also reported that the bioengineered tooth germ has the potential to erupt from a region of a lost tooth in mice [194]. It also had the potential to be transplanted [198], providing a technique that brings regenerative medicine one step closer to whole-tooth replacement [192].

The method of how to produce organoids was described first in 2008 [199]. To do so, an organ-inducing field is mimicked on a cell aggregate of stem cells, which will induce the stem cells to self-organize. However, stem cells in vitro are only capable of partially reproducing structures of an organ and achieve only limited sizes; hence, they are also often termed “mini-organs” [192]. The organ-inducing field is achieved by applying cytokines to recapitulate patterning and signaling in the embryo [192]. Depending on which questions will be addressed, organoids can be derived from pluripotent stem cells (PSCs) and adult stem cells (ASCs). The choice of cell type for organoid formation influences the mechanisms involved in organoid formation, as these cells arise from different developmental stages [200]. ASCs play a role in homeostasis or regeneration of an organ in the organism; hence, using ASCs might recapitulate these processes mimicking the stem cell niche. While in PSCs, the organ-inducing field provokes an organization similar to what occurs in embryonic organogenesis and hence provides information about developmental processes [192,200]. The choice of cells also largely determines the maturity of the organoid that can be reached in vitro, with organoids from PSCs mimicking the immature tissues [200]. Organoids find applications in basic biology, in understanding developmental processes or processes of regeneration and homeostasis, but they also are valuable for disease modeling. They can be also obtained from patient-derived stem cells; hence, they also provide a great resource for drug screening or personalized medicine and could also be applicable for regenerative medicine approaches in the future [200]. Unlike bioengineered organ germs, organoids are not capable of completely obtaining the functions of the original organ if transplanted by themselves, while multiple organoids transplanted together may restore partially the organ function [192].

**Figure 3:**
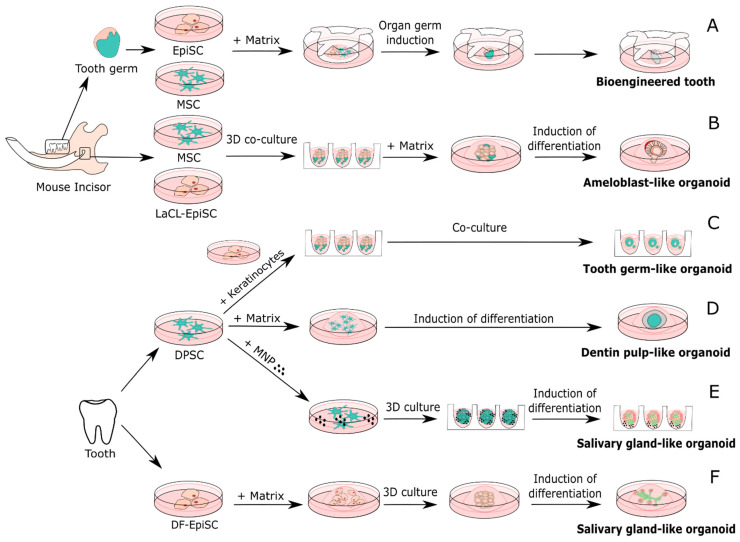
Organoids and organ germs from dental tissues. (**A**) Scheme of the “organ germ method” for the generation of a bioengineered tooth, with cells derived from a tooth germ, cultured in compartments in collagen, inducing the formation a tooth germ in vitro and finally give rise to a bioengineered tooth [193]. (**B**) Scheme of the generation of an ameloblast-like organoid from EpiSCs in the presence of dental mesenchyme, cultured in non-adherent plates, and further propagated in Matrigel with inducing factors [206]. (**C**) Scheme of the generation of tooth germ-like organoids from DPSCs in the presence of keratinocytes in a low-adherent culture [207]. (**D**) Scheme of the formation of dentin pulp-like organoid from DPSCs [208]. (**E**) and (**F**): Scheme of the formation of salivary gland-like organoids by different methods and cells: (**E**) Organoid formation from DPSCs using magnetic nanoparticles and low attachment conditions [209], (**F**) Organoid formation from DF-EpiSCs in a Matrigel matrix [210].

One of the earliest published studies in the field of organ-like regeneration from dental tissues was by Nakao and colleagues, who constructed a tooth-like structure from injecting isolated and dissociated epithelial and mesenchymal cells into a collagen matrix, which they cultured in vitro and transplanted into mice (Figure 3). They described the formation of different compartments and tissues (including odontoblasts, dentin, ameloblasts, and enamel) in their bioengineered tooth [193]. The relationship of the contact area between mesenchymal and epithelial cells later was identified to be determining for the crown width of the bioengineered tooth and was found to be related to cellular proliferation due to Sonic hedgehog signaling [201]. IGF-1 was found to increase the size of the tooth, and a combination with BMP2 could improve odontoblastic differentiation in that model [202]. Tooth germ-like structures can also be obtained by using dental mesenchymal immortalized cell lines instead of mesenchymal tissue of tooth germs, as shown in further studies [203]. Similarly, it was shown that mouse iPSCs addition to the mesenchymal and epithelial compartment culture, and growth in the subrenal capsules of mice, allowed bone or dentin-pulp like structure formation [204]. The major limitation of this method was its efficacy. Bioengineered tooth generation was often unsuccessful. Thus, the epithelial and mesenchymal cells were derived from embryonic tooth germs, which is a source not practicable in humans. More recently, it could be shown that a bioengineered tooth, functionally replacing a tooth loss in a large animal model, could also be derived postnatally from deciduous teeth or permanent tooth germs. Additionally, in the canine model, the generation of a bioengineered tooth had a low efficiency when dissociated mesenchymal or epithelial cells were used in comparison to using mesenchymal or epithelial tissue directly [205]. In young patients, tooth germ material may be easily obtained from deciduous teeth or wisdom teeth, which proposes a realistic alternative. However, this age group is usually not the one posing a large demand on tooth replacement. iPSCs may provide an alternative source in the future for this kind of organ regeneration [205].

DSCs were used as a starting material to construct tooth germ-like [206], dentin pulp-like [207], ameloblast-like [208] and salivary gland-like organoids [209,210]. One of the first steps in tooth development in the embryo is the formation of an ectomesenchymal condensate, which forms the tooth bud [206]. This condensation can be modeled from human DPSCs, which are cells of ectodermal origin with a mesenchymal phenotype that undergo self-organization together with epithelial cells. Rosowski and colleagues reported cell–cell attachment as first part of the organization and an interaction of DPSCs with gingival keratinocytes (adult epithelial cells), thus showing the potential of this tooth germ-like organoid to recapitulate major steps of tooth development (Figure 3). However, there is still room for further investigations—most importantly, the choice of epithelial cells [206]. Recently, it was shown that DPSCs were capable of differentiating into odontoblasts in a self-organized organoid by giving the right stimuli (Figure 3). Dentin pulp-like organoids retained stem cells, providing a better maintenance of the organoids as shown by the dissociation and reassembly of the organoids [207].

Much less investigated than DSCs are dental epithelial stem cells (EpiSCs). The EpiSCs’ sphere-forming ability was previously reported. The composition of dentospheres from these cells was largely influenced by culture conditions [189]. In mice, EpiSC continue to differentiate into ameloblasts, producing enamel. In humans, enamel is produced only once, when the tooth crown is formed. In a 3D culture system with mesenchymal and epithelial compartments from cells derived from the labial cervical loop (LaCL) of mice, organoids containing ameloblasts and preameloblasts were reported (Figure 3) [208]. However, matching studies from human cells are still lacking. One of the limitations of such studies with EpiSC is their limited availability in humans due to the lack of HERS/ERM after tooth eruption [186].

The generation of the salivary gland-like organoids was shown (Figure 3); these organoids contained acinar, ductal, and myoepithelial cells [209,210], but also a neuroepithelial compartmented and secretory epithelium from DSCs [209]. For the formation of salivary glands using dental stem cells, EpiSCs isolated from the dental follicle (DF-EpiSCs) were used, while in another study DPSCs showed similar gland formation capacities [209]. The salivary gland-like organoids also showed the capability to induce the growth of epithelial cells in an ex vivo irradiated salivary gland model upon transplantation [209]. The organoids secreted amylase [209,210]. They also responded to neurotransmitters, indicating a neuronal network [209]. The spheroid formation of DPSCs using magnetic particles via the magnetic 3D bioprinting (M3DB) system is an alternative scaffold-free bioengineering technique to achieve 3D arrangement of cells [209]. Taken together, stem cells isolated from dental tissues could provide a promising alternative for salivary gland tissue engineering, using an easily accessible source of stem cells for that matter [209,210].

The major limitation of both organ-resembling methods namely remains proper vascularization. The lack of it provokes necrosis, which is crucial to achieve a culture of sufficient size for organ replacement [192]. Recently, a vascularization network was shown in spheroids [211], providing a promising outlook for regenerative medicine application such as whole organ replacements [192]. There have been many advances in the field of organoids and organ bioengineering in the recent years, and they still hold great promise; however, also, many questions remain unsolved. Still, proper innervation, mechanical cues, and immune responses in these organ replacements are problems that remain to be tackled [212]. This research is also proceeding by improving other organ culture platforms. One direction is a “microfluidic organ-on-chip” platform, with different compartments, which is providing mechanical cues by trying to resemble complex organ-typic physiological processes [213].

**Figure 4:**
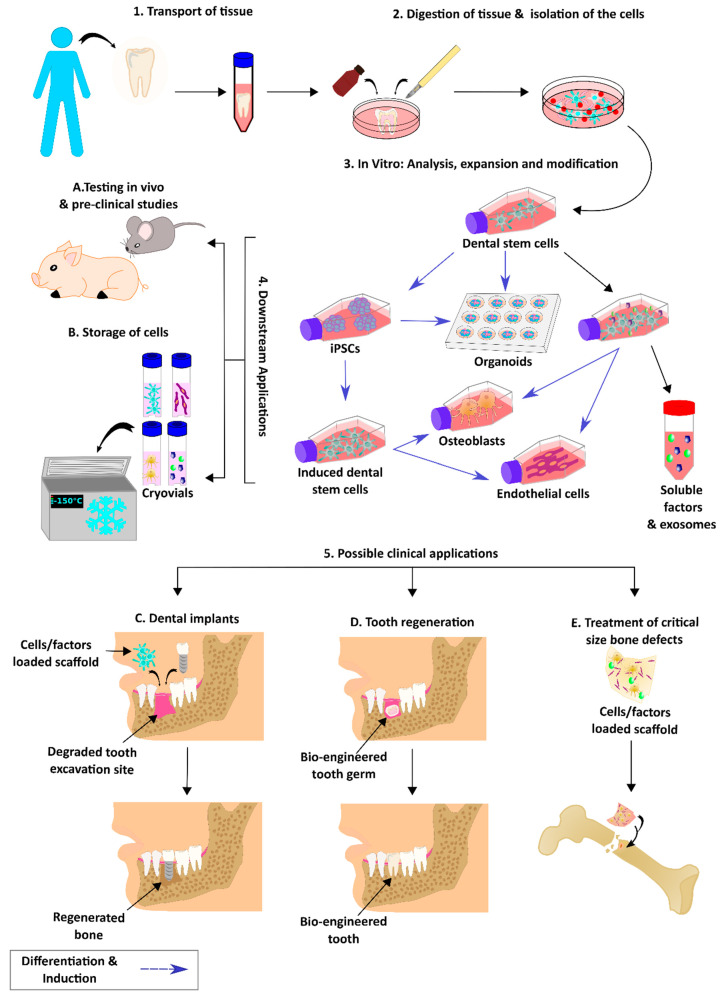
Possible applications of dental stem cells in research and therapy. Dental tissues are acquired from the patient (**1**) to isolate the dental stem cells (**2**); then, the DSCs can produce differentiate cell types (**3**), DSCs also produce soluble factors and exosomes. Then, the factors and exosome are characterized and tested in vivo (**A**), after which they are cryopreserved for future use (**B**) in (**4**) possible future therapy applications (**5**) such as for improving dental implants (**C**), whole tooth regeneration (**D**) and treatment of sever bone injuries like critical size bone defects (**E**).

The generation of immortalized cell lines, iPSCs, and organoids has many applications in research in vitro and in vivo, and possibly in future therapy. However, the use of stem cells in therapy is a long-standing approach. In tissue regeneration, there are multiple pillars that are considered to be vital for its success: the right choice of cells, a biomaterial scaffold for the cells to grow in 3D providing suitable biophysical and chemical signals to recapitulate the tissue, which is termed the tissue engineering triad [214,215]. In the next sections, these three pillars will be further discussed, with respect to applying DSCs in stem cell therapy for bone tissue restoration. Figure 4 describes some examples of the research and possible therapy applications using dental stem cells.

## 3. Regulators and Enhancers of Osteogenesis in Dental Stem Cells

Bone repair is a series of complex physiological events including inflammatory, chondrogenic, osteogenic, and angiogenic events whose interplay is vital [216,217,218]. Stem cells are one of the crucial cellular elements in bone repair [219]. It is well known that dental stem cells are capable of osteogenesis, differentiating to osteoblasts and odontoblasts [21,220,221,222]. In this chapter, growth factors and environmental regulators that could enhance the dental stem cells’ innate osteogenic regenerative potential will be introduced.

Stem cells derived from different dental and other tissues have been compared for their potential for bone tissue regeneration. However, the reports are inconsistent. In some studies, it was reported that stem cells from dental tissue are similar or even better to than other stem cells for osteogenic differentiation [17,58,223,224,225]. When DFSCs and adipose tissue derived MSCs (A-MSCs) were differentiated toward osteoblasts in vitro, DFSCs showed a better mineralization [17]. In 2012, Rho and colleagues reported that DFSCs, BM-MSCs, and skin-derived MSCs can be osteogenically differentiated in vitro without the addition of a specific osteogenic media when cultured on a demineralized bone matrix or a fibrin glue scaffold [223]. When the three stem cell-based scaffolds were implanted with scaffolds in vivo, the scaffolds loaded with DFSCs showed the highest osteocalcin (OCN) expression and calcium deposit [224]. Szepesi and colleagues compared the regenerative potential of PDLSCs, A-MSCs, and Wharton’s jelly-derived MSCs (WJ-MSCs) in vitro [223]. PDLSCs were comparable to A-MSCs in relation to their osteogenic and angiogenic potential in vitro, with both stem cell types showing stronger osteogenic and endothelial differentiation than MSCs isolated from Wharton’s jelly [223]. While SHED are also isolated from dental pulp from deciduous teeth, DPSCs are from permanent teeth. Therefore, SHEDs are considered more immature than DPSCs. Other differences have been shown in patterns of gene expression and proliferation and differentiation potential between DPSCs and SHED [225,226,227,228]. Nakamura and colleagues compared the gene expression profile of SHED, DPSCs, and BM-MSCs, showing that SHED had a higher capacity for extracellular matrix production and proliferation [225]. In another study, the bone regenerative potential of BM-MSCs, SHED, and DPSCs was compared for the treatment of an artificial calvarial bone defect in vivo [58]. SHED and DPSCs were able to form new bone in the defects similar to BM-MSCs after transplantation on a poly(lactic-co-glycolic acid) (PLGA) membrane. The SHED seemed to have the highest percentage of collagen and osteoid area formation and bone regeneration rate among the groups, albeit this difference was not significant [58].

However, in other studies, another trend was shown [74,229,230]. DPSCs and SHED were compared to BM-MSCs and synovial fluids-derived stem cells (SF-MSCs). An in vitro investigation of osteogenesis and chondrogenesis showed that BM-MSCs and SF-MSCs had better differentiation potential than SHED and DPSCs [229], with SHED having a slightly better osteogenic capability than DPSCs. In 2019, Jin and colleagues compared A-MSCs and DPSCs in vitro and in vivo. Their results in vitro also showed that DPSCs have a higher colony formation and proliferation capacity in comparison to A-MSCs [74]. They demonstrated also that A-MSCs exhibited a stronger osteogenesis compared to DPSCs in vitro [74,230]. This was also apparent in vivo in a rat mandibular defect model, where A-MSCs showed better osteogenesis and mineralization at week 1 after implantation comparing to the DPSCs after 3 weeks [74].

In 2018, stem cells isolated from seven different tissues were compared, including four from dental and oral origins (DPSCs, PDLSCs, G-MSCs, and DFSCs), BM-MSCs, A-MSCs, and MSCs from the umbilical cord (UC-MSCs) [230]. While the cells were cultured and differentiated under the same conditions, the dental stem cells and UC-MSCs proliferated better than BM-MSCs and A-MSCs. Regarding the osteogenic potential, they reported that BM-MSCs and A-MSCs had the highest ALP activity followed by PDLSCs and DFSCs, whereas UC-MSCs and G-MSCs rarely differentiated within conditions they used. The mineralization was also the highest in A-MSCs, followed by BM-MSCs and PDLSCs and DFSCs. From the dental stem cells, DPSCs had the lowest ALP activity and mineralization.

The differences between reports can have multiple explanations. The function and differentiation potential can be affected by multiple factors during cell isolation and culture conditions [231], by donor age [232,233], by the tissue-harvesting sites (e.g., mature wisdom teeth or exfoliated teeth) [234,235], and osteogenic induction methods. Still, in general, stem cells derived from dental tissue are still interesting for bone regeneration. Even if their osteogenic potential might not be as good as A-MSCs or BM-MSCs, they still have many advantages. One advantage is that acquiring dental tissues can be less invasive compared to BM-MSCs, as they are “medical waste”, which makes them ethically less problematic. Moreover, they possess a higher proliferation capacity compared to other stem cells, which makes expanding them in vitro easier.

However, the innate differentiation and proliferation ability of stem cells are not the only factors that weigh into their value for tissue regeneration. Differentiation and proliferation can be triggered and improved through physicochemical and mechanical cues [236]. In their physiological microenvironment, stem cells are subjected to various soluble and paracrine parameters, which promote their self-renewal and tissue regeneration abilities [237]. In the next subsections, research on the improvement of DSCs’ osteogenic potential with a focus on growth factors and other environmental cues will be reported.

### 3.1. Growth Factors Regulation of Osteogenesis in Dental Stem Cells

It was shown that the differentiation of stem cells toward osteoblasts depends on numerous signaling pathways and that the process itself could be enhanced and regulated by certain growth factors [8,238,239,240,241,242]. Some growth factors are already approved by the FDA as drugs for treatment, such as the recombinant human bone morphogenic protein 7 (rhBMP7) in 2001 or the rhBMP2 in 2002 [243,244].

Two major groups of regulators of osteogenesis in stem cells are the transforming growth factors-beta (TGF-β), namely TGF-β1 and TGF-β2, and bone morphogenic proteins (BMPs), BMP2, BMP4, BMP6, BMP7, and BMP9 [245]. BMPs also belong to the superfamily of transforming group factors-beta [246]. The overexpression of TGF-β1/2 or of the mentioned osteogenic BMPs induced bone formation in vitro as well as in several animal studies in vivo [247,248]. Treatment of differentiating MSCs with BMPs increased the expression of osteoblast-specific markers, including the early marker Runt-related factor-2 (Runx-2), and the late markers OCN and OPN, which were upregulated specifically by BMP2 [249,250,251]. In addition, it was shown that BMPs improve the osteogenic differentiation of MSCs by increasing ALP activity and matrix mineralization [252]. Similar results were observed with TGF-β1, which is one of the most abundant cytokines in the bone matrix [253]. Another regulatory growth factor family, which plays a major role in the osteogenesis of stem cells, is the fibroblast growth factor (FGF) family, containing 22 members [254]. The major FGFs that play a role in the process of osteogenesis are FGF2 (bFGF), FGF9, and FGF18. FGF2 was described as a positive regulator of bone mass [255], while additional FGF9 treatment showed an improved bone healing in vivo [256]. The function of FGF18 was described as a promotion of osteoblast differentiation [257].

Other growth factors known to regulate the osteogenesis of stem cells are insulin and insulin-like growth factor-1 (IGF-1) [258]. The binding of insulin to its receptor activates a downstream pathway, which enhances osteogenic differentiation and the activity of osteocytes [259,260]. Insulin and IGF-1 exposure to differentiating mesenchymal stem cells is described to increase the expression of bone synthesis markers, collagen synthesis, and ALP production [258]. The vascular endothelial growth factor (VEGF) is vital for the coupling of angiogenic and osteogenic processes during both skeletal development and bone repair [261,262]. VEGF is used mostly in combination with other growth factors such as BMP2 sharing the downstream signaling pathways. Rarely used growth factors such as the stromal-derived factor-1α (SDF-1α) [263] or platelet-derived growth factor (PDGF) [264] also play a critical role in regulating the osteogenesis of stem cells. SDF-1α was described to be induced in SCAP by BMP2. The blocking of SDF-1α affected BMP2-induced ALP activity and the expression of RUNX2 significantly [264]. PDGF was depicted to increase osteogenic differentiation of mesenchymal stem cells but decrease adipogenic differentiation via the extracellular signal-related kinase 1/2 (ERK1/2) transduction pathway [265].

All of the above-mentioned growth factors also regulate the osteogenic differentiation of DSCs. Table 1 summarizes the factors mentioned and the effect they exert on osteogenesis. In the next section, the role of growth factors will be discussed for DPSCs (including SHED and adult DPSCs), PDLSCs, and DFSCs.

#### 3.1.1. Regulation of Growth Factors in Osteogenesis in Dental Pulp Stem Cells

Osteo-promoting effects have been described for IGF1 [266,267,268], for VEGF [266,269,270], and for TGF-β1 [271,272] in DPSCs. Previous findings on bFGF/FGF2 were more contradictive, mostly depicting an inhibitory role [273,274,275,276,277]. A combination of bFGF with TGF-β1 in SHED was described to enhance osteogenesis [271], while others showed the promotion of osteogenesis if it is delivered at the right time point during differentiation [278,279,280]. An inhibitory role on osteogenesis in DPSCs was noted with FGF9 [281]. There is also a disagreement on BMPs effects on osteogenesis in DPSCs. A promotion of osteogenesis via BMP2 was reported in SHED [282,283,284,285] and DPSCs [283], while others did not find effects of BMP2 on osteogenesis or odontogenesis by itself [269,286]. Recently, osteo- and odonto-promotive properties of BMP7 and BMP9 were described for DPSCs [287,288]. From studies of MSCs, it was shown that IGF1 is capable of enhancing osteogenesis and proliferation. IGF1 was described to have benefits in cellular proliferation and the induction of osteogenesis and odontogenesis in DPSCs [267,268] and CDPSCs [266]. The effect on osteogenesis occurs most likely via mTOR activation and PI3K/Akt signaling [268]. In relation to this, Lv and colleagues reported an enhanced phosphorylation of also ERK1/2 and p38 in response to IGF1 treatment, pinpointing the role of these kinases in IGF1-mediated differentiation [267]. It was previously described that ERK may converge with the PI3K/Akt pathway and that osteogenesis can be regulated via MAPKs [289]. Lu and colleagues showed that in CDPSCs, a combination of treatment with two growth factors IGF1 and VEGF could enhance proliferation, migration, and osteogenic differentiation better than any of them separately [266]. Zhang and colleagues showed that the stable overexpression of VEGF in DPSCs upregulated odontogenic markers and increased ALP and OCN expression [270]. Aksel and Huang showed that VEGF enhanced osteogenesis and mineralization, especially if the stimulation was done at the beginning of osteogenesis [269]. Stimulation with VEGF for 1 week and BMP2 throughout the differentiation process enhanced osteogenesis to a lesser extent, which was possibly because of a downregulation of BMP2 by VEGF [269]. Similarly, Lu and colleagues could not only show an increased expression of osteogenic markers in response to VEGF treatment but also the proliferative and migrative abilities of CDPSCs, which were more pronounced in combination with IGF1 [266]. In another study, a positive effect of EGF on calcium deposition and osteogenic marker expression was reported in DPSCs, which was similar to the effect on BM-MSCs [273].

FGFs were also tested for their effect on DPSCs. Data from in vivo studies depicted an inductive effect of bFGF on bone regeneration [279], while in vitro studies using DPSCs remain controversial. He and colleagues were some of the first exploring the effects of FGFs in DPSCs. BFGF was shown to have a positive effect on the proliferation of DPSCs, while its effect on cellular differentiation was not pronounced. No mineralization was observed in the presence of bFGF alone, but a strong mineralization was seen with a combination of bFGF and TGF-β1. This indicates a promotion of bFGF on the effect of TGF-β1 during the differentiation [271]. Kim and co-workers reported an increased odontogenesis in DPSCs in response to bFGF through PI3K/Akt/NF-κB signaling [278]. Novais and colleagues reported enhanced bone healing with SHED primed with bFGF before engraftment that resulted in an increased secretion of VEGF and hepatocyte growth factor by SHED. They also noted earlier proliferation and stronger mineralization, both in vivo and in vitro, in response to bFGF [280]. On the other hand, Li and colleagues reported decreased MSCs marker expression and osteogenesis in SHED and reported the MAPKs as downstream targets of bFGF treatment. Using a sufficiently high concentration of bFGF, they observed activation of ERK1/2. Inhibition of ERK1/2 could restore the mineralization ability and β-catenin production in these cells. Overall, they suggest that the negative regulation of bFGF is mediated via activating Ras-Raf-ERK, which then regulates the Wnt pathway by reducing β-catenin/active β-catenin production [276]. Nowwarote and colleagues investigated the relationship of bFGF and the regulation of phosphate/pyrophosphate (Pi/PPi) in the mineralization and osteogenesis of SHED. In an early study, they reported an attenuation of ALP in response to bFGF and reduced osteogenesis, indicating a negative role of bFGF on osteogenic differentiation [275,277]. In response to bFGF, they observed a downregulation of mRNA of Notch receptors, ligands, and targets. Hence, they propose that the negative effect on osteogenesis is mediated via the bFGF receptor and MAPK/ERK kinase signaling, while the observed effect could be reversed by addition of the Notch ligand, JAGGED1 [277]. They also found that Foscarnet, which blocks the uptake of Pi, could dramatically decrease osteogenesis, while ALP generates Pi, which was shown to promote osteogenic differentiation [277]. In further studies, they observed that bFGF leads to a decrease in the Pi/PPi ratio. The addition of PPi inhibited mineralization and osteogenic-related genes, while the addition of Pi provoked increased mineralization and upregulated osteogenic-related genes; this is in line with the importance of Pi and PPi previously described in mouse models [274]. Consistent with this is the data reported by Del Angel-Mosqueada and colleagues, that bFGF had an inhibitory effect on osteogenic differentiation in DPSCs [273]. Qian and colleagues tried to shed light onto the inconsistent findings following bFGF treatment in MSCs by investigating the role of bFGF under different conditions. In Vitro and in vivo, they found a decrease in osteogenesis when the cells were stimulated with bFGF during the osteogenic induction period or during a 2-week pretreatment. However, they described increased osteogenesis both in vivo and in vitro when cells were pretreated for 1 week only [279]. The differences in effects of bFGF point to different roles of bFGF signaling in the time course of osteogenesis [279]. Another member of FGFs, FGF9, was described to negatively regulate osteogenesis in both, DPSCs and BM-MSCs, where it was found to increase phosphorylation and thus activated ERK1/2, which is a regulatory mechanism [281].

During the mineralization process in DPSCs, TGF-β-related genes are differentially expressed, indicating a role of TGF-β signaling in these cells [290]. In line with this, TGF-β1 was reported to stimulate the differentiation of DPSCs toward osteoblasts, as shown by ALP expression, morphology, and mineralization. This effect was strongest with a combination of TGF-β1 and FGF2, suggesting synergic effects [271]. TGF-β was also found to be upregulated by SHED in response to hydroxyapatite, similar to RUNX2, pointing to both molecules as indicators for osteogenesis [291]. In agreement with this, Farea and colleagues showed that the cells differentiating on the chitosan scaffold and treated with TGF-β1 showed the highest ALP levels and mineralization from their tested combinations [272].

As mentioned before, other important growth factors in osteogenesis are BMPs. Data on the effect of BMP2 in DPSCs are rather inconsistent. Koyoma and colleagues previously showed that BMP2 treatment triggers osteogenesis in both DPSCs and SHED, even in the absence of osteo-inductive medium, indicating its strong osteo-promotive role [282]. Similarly, it was reported by other groups that BMP-related genes were increased during osteogenesis and that BMP2 could upregulate osteogenic and odontogenic markers [283]. In addition, the inhibition of BMP2-neutralizing antibodies diminished osteogenesis in SHED [284]. On the other hand, Aksel and Huang reported not much difference in mineralization when BMP2 was added to osteogenic medium compared to the medium alone. However, they observed increased osteogenesis when VEGF was added in the first 7 days during the differentiation process. Treatment with only BMP-2 at different durations or in combination with VEGF exhibited a weaker induction of osteogenesis [269]. In addition, in DPSCs transfected with an inducible BMP2 transgene overexpressing BMP2 upon doxycycline stimulation, no increased osteogenesis was observed [286]. Interestingly, next to an overexpression of BMP2, an increased expression of noggin, a BMP antagonist, was observed, which could potentially compensate for the effect of BMP2 [286]. The different effects of BMP2 described might be time point related but also result from different concentrations of BMP2s applied [269,283]. In addition to BMP2, BMP9, and BMP7 are promising agents involved in osteogenesis and odontogenesis [287,288]. Li and colleagues showed that the expression pattern of BMP9 was higher in the odontoblast layer compared to the central area of the pulp, indicating a possible role in dentin formation. By overexpressing the BMPs, they observed a higher odontogenic potential with BMP9 even better than that of BMP2 both in vitro and in vivo [287]. BMP9 promoted odontogenesis via increased phosphorylation of MAPKs (JNK, p38, ERK1/2) [287]. Zhu and colleagues showed osteo-inductive properties of BMP7 on DPSCs by upregulating odontogenic/osteogenic markers and mineralization in a dose-dependent manner. They observed an increased phosphorylation of Smad5 and BMPR1A/ALK3 expression as downstream targets in odontogenesis [288].

#### 3.1.2. Regulation of Growth Factors in Osteogenesis in Periodontal Ligament Stem Cells

PDLSCs are involved in the regeneration and maintenance of the periodontal ligament, as well as alveolar bone and cementum. The treatment of PDLSCs with members of BMP family was shown to have a positive effect on osteogenesis in this cell type as well. PDLSCs that were genetically modified to produce a sustained release of BMP2 had improved osteogenesis in vitro and in vivo [105]. BMP7 was also shown to improve osteogenesis in PLDSCs. Acil and colleagues showed that BMP7 treatment upregulated the expression of RUNX2 and OCN in a dose- and time-dependent manner [292]. Wang and colleagues were able to enhance the osteogenic potential of PLDSCs by transfecting them with BMP9. Moreover, the combination of BMP9 transfection and pulsed electromagnetic field treatment improved the osteogenesis of these cells depicted by an enhanced expression of RUNX2, ALP, and OPN [293]. Ye and colleagues reported that the effect of BMP9 on osteogenesis is mediated through the activation of the MAPK signaling pathway via the phosphorylation of p38 and ERK1/2. The expression levels of osteogenic markers were reduced when the p38 signaling pathway was inhibited, while they increased when ERK1/2 was inhibited [294]. Stimulation with either BMP2, BMP6, or BMP7 improved osteogenic differentiation. Notably, treatment with BMP6 resulted in the highest mineralization [295]. Treatment of PDLSCs with VEGF was shown to promote the mineralization and expression of osteogenic markers [296]. Another growth factor, IGF-1, was shown to improve the proliferation and osteogenic capabilities of PDLSCs in a dose-dependent manner in vitro. The transplantation of PDLSCs on IGF-1-treated implants into renal capsules of mice improved the mineralization and higher expression of RUNX2, osterix (OSX), and OCN compared to the control [297].

Contrarily, treatment of PDLSCs with bFGF was shown to promote proliferation and migration, but it inhibited osteogenesis in multiple reports, even when combined with BMP2 and BMP4 [296,297,298]. A study showed that the consecutive treatment for 3 days with bFGF followed by treatment with BMP2 for the duration of the differentiation resulted in increased osteogenic differentiation [298].

#### 3.1.3. Regulation of Growth Factors in Osteogenesis in Dental Follicle Stem Cells

Numerous growth factors have been described to be involved in the osteogenic differentiation process of DFSCs. TGF-β2 was described to downregulate the osteogenesis of dental follicle stem cells [299]. Contrary to that, factors such as BMP2 [300] through NOTCH signaling [301], BMP6 [302,303], and BMP9 [304] were described to induce the osteogenic differentiation of DFSCs. Um and colleagues compared the osteogenic differentiation of inflamed and normal DFSCs. The osteogenic differentiation of inflamed DFSCs showed a decreased ALP activity and Alizarin red S staining compared to the normal stem cells. In line with this, the transplantation of inflamed DFSCs into dorsal skin of mice led to a severe impairment of osteogenesis compared to normal DFSCs. After an analysis of the protein profile of both DFSCs types, significant changes in the expression level of TGF-β1 and TGF-β2 were observed. TGF-β1 levels were lower and TGF-β2 levels were higher in inflamed DFSCs. The inhibition of TGF-β2 resulted in increased levels of TGF-β1, ALP activity, and mineralization [299].

The osteogenic differentiation of DFSCs induced by BMP2 occurs through the canonical Wnt/β-catenin pathway. Silvério and colleagues suggested that the Wnt/β-catenin pathway induced by the wingless-type MMTV integration site family member 3A (WNT3A) is a key player in this pathway in DFSCs. The activation of Wnt/β-catenin signaling with WNT3A suppressed the BMP2 mediated induction of osteoblasts of DFSCs and was also responsible for a downregulation of RUNX2, ALP and OCN [300]. Another example of the influence of BMP2 on the osteogenesis of DFSCs was shown by Viale-Bouroncle and colleagues. BMPs were described to be supported by the transcription factor distal-less homeobox 3 (DLX3) via a positive feedback loop. The group elucidated an additional pathway to the BMP2/DLX3 pathway by the overexpression of DLX3 and the induction of osteogenesis in DFSCs with BMP2 and dexamethasone. Under these conditions, the NOTCH signaling pathway was activated, which also led to ALP activity and mineralization [301]. They concluded that the NOTCH signaling pathway regulates the BMP2/DLX3-directed differentiation via a negative feedback loop in DFSCs [302]. In addition to BMP2, also BMP6 expression in DFSCs corresponds with osteogenesis. It was shown by Yao and colleagues that BMP6 expression was significantly higher in dental follicle stem cells than in their non-stem cell counterpart, dental follicle cells (DFCs). It was shown that DFSCs lost their osteogenic capability in vitro after expansion and cultivation in late passages (p7-p9), due to reduced BMP6 expression as compared to early passages (p3-p5). Supplementation with exogenous BMP6 during the osteogenic differentiation of late-passage DFSCs significantly enhanced osteogenesis, while a knockdown of BMP6 with RNAi during the differentiation of early-passage DFSCs decreased the osteogenesis. This effect was restored by the addition of BMP6 [303]. Takahashi and colleagues were also stating that BMP6 plays an important role during the osteogenesis of DFSCs. They found that BMP6 enhances the gene expression level of RUNX2 and other osteogenic markers. Additionally, the mineralization was stimulated by BMP6, and therefore, BMP6 was described as a key gene that has to be expressed in high levels to maintain the osteogenesis capability of DFSCs [304]. Another bone morphogenic protein that was described to play an important role in the osteogenesis of DFSCs is BMP9. Li and colleagues isolated DFSCs from rat dental follicles. These cells were transfected with a BMP9 containing vector and differentiated toward osteoblasts. In comparison to the non-transfected cells, ALP activity and calcium deposition were significantly increased. The BMP9-induced osteogenesis of DFSCs was regulated via p38 MAPK and ERK1/2 [305].

### 3.2. Environmental Stimulators Regulating Osteogenesis in Dental Stem Cells

Stem cell differentiation and function is influenced by chemical, mechanical, and physical microenvironmental cues in the body; this is also part of the stem cells niche [306,307]. Therefore, understanding the effect of these stimulators on stem cells could possibly allow a better control and improvement of their function by preconditioning stem cells in vitro before using them in vivo for clinical applications.

One of these stimulators is hypoxia, which refers to low oxygen conditions. Hypoxia is mediated mostly by hypoxia-inducible factors (HIFs), and it plays an important role in bone as a driving force for coupling angiogenesis and osteogenesis during bone repair and development. In MSCs, hypoxia can increase the expression of genes such as those involved in glycolysis and angiogenesis [308,309,310,311,312,313]. It was also shown to affect osteogenesis in DSCs. Culturing DFSCs in a hypoxic chamber for 7 days enhanced their proliferation and osteogenic potential in vitro [314]. Li and colleagues investigated the effects of hypoxia on PDLSCs, and they found an increased proliferation and expression of the osteogenic markers OPN, RUNX2, and OCN after culturing the cells in hypoxic conditions for 7 days in vitro and higher osteogenesis in vivo under the same conditions [315]. They also reported increased expression of RUNX2 and OSX during osteogenic differentiation under hypoxia compared to the undifferentiated control and cells under normoxia [316]. Hypoxia was also reported to increase the release of prostaglandin E2 and pro-angiogenic VEGF from PDLSCs. It was revealed that this effect is mediated through MEK/ERK and p38 MAPK signaling, which is known to be crucial for the osteogenic differentiation of MSCs [317,318]. Moreover, MAPKs were shown to mediate HIF-1 dependent VEGF expression, which can explain the increased levels of secreted VEGF in PDLSCs [319]. The phosphorylation of ERK1/2 showed a slow and constant pattern, while on the other hand, p38 responded to hypoxia more rapidly and vigorously. In another study, it was shown that 24 h hypoxia pretreatment increased migration and cell CXCR4 expression and enhanced RUNX2 and ALP protein expression during the differentiation [320]. The activation of the receptor CXCR4 by its binding protein SDF-1 is associated with the regulation of stem cell mobilization and migration [321] as well as subsequent migration to an injury site [322,323]. The recruitment of stem cells by SDF-1 was shown to promote bone repair [324]. SDF-1 expression was described to be regulated by HIF-1α in BM-MSCs, resulting in increased migration to hypoxic sites as well [325]. Contrary to this, stem cells that are expanded in vitro showed CXCR4 downregulation [326]. Taken together, hypoxia pretreatment seems to be a suitable option to enhance CXCR4 expression and subsequently stem cells migration and differentiation in vivo [320,327]. However, this enhancement is dependent on multiple mechanisms and conditions, which still need to be better clarified; as in other studies, it was shown that hypoxia and an increased expression of HIF-1 could decrease osteogenesis. In a 2015 study, the induction of hypoxia using cobalt chloride (CoCl_2_) was shown to have no effect on the proliferation of a heterogeneous population of PDLCs [328]. Moreover, adding CoCl_2_ to PDLCs during osteogenic differentiation suppressed it, possibly through HIF-1α. Deng and colleagues reported that PDLSCs differentiated under hypoxic conditions resulted in decreased osteogenesis. They also showed that TGF-β1 was responsible for the stabilization of HIF-1α, and the stimulation of PDLSCs with TGF-β1 under hypoxic conditions resulted in a stronger decrease in osteogenesis [329].

HIF-1 and TGF-β1 seem to have a dual effect on osteogenesis through multiple mechanisms, depending on the stages of differentiation [330]. TGF-β1 can enhance stem cells’ commitment to the osteogenic lineage by reorganization of the actin cytoskeleton [331]. HIF-1 can facilitate the induction of β-catenin, enhancing the osteogenesis of BM-MSCs by inhibiting miR-340-5p [332]. However, TGF-β1 can also inhibit osteoblastic differentiation by antagonizing BMP2 through inhibiting the activation of Smad1, 5, and 8 [333]. In BM-MSCs, hypoxia can result in the inactivation of RUNX2 via the ERK1/2 and p38 MAPK signaling pathways, resulting in decreased osteogenesis [334].

Another environmental stimulator, light, is important on a systemic level for circadian rhythms and the processing of vitamins, among other roles. Low-level laser therapy (LLLT) or photobiomodulation was first discovered by accident when the Hungarian physician Endre Mester tried using it as treatment for tumors only to find that it resulted in stimulating hair regrowth and wound healing in rats [335]. Since then, it was proven to be an effective biostimulant and was investigated for multiple application including bone regeneration [336,337,338,339,340,341,342,343,344,345,346] and angiogenesis [347,348,349,350]. The effect of photobiomodulation was also studied in DSCs. Turrioni and colleagues investigated the effect of irradiation on SHED by an infrared LED (850 nm) with respect to dentin matrix expression and synthesis [351]. They observed an increase in ALP activity and collagen synthesis and an increased gene expression of DSPP and ALP in the treated cells. In 2019, Paschalidou and colleagues investigated the effects of low-level laser irradiation (LLLI) on the proliferation, migration, and osteogenic differentiation of SHED [352]. They defined the range with the highest effectiveness on the induction of osteogenesis. In general, the cells treated with LLLI had an upregulation of BMP2, OCN, DSPP, and RUNX2 without osteogenic induction, and cells treated with LLLI and osteogenic induction depicted a higher increase in the gene expression of ALP, BMP2, OCN, DSPP, and Msh homeobox 2 (MSX2). LLLI seems to have a positive effect on stem cells seeded in 3D agarose gel, as shown by Kopper and colleagues Another form of phototherapy, high-power red LED irradiation, increased proliferation and ATP levels during the differentiation of PDLSCs [353]. It also resulted in enhanced osteogenesis seen by the increase in ALP activity, OCN production, and enhanced mineralization. This was accompanied by an upregulation of RUNX2 and OSX gene expression due to the activation of ERK1/2, which was enhanced with LED irradiation. Diniz and colleagues examined the combined effect of photobiomodulation and BMP4 on the osteogenesis of DPSCs in vitro and in vivo. They encapsulated DPSCs in an injectable and thermoresponsive cell carrier loaded with rhBMP4 and then photoactivated it. This treatment enhanced mineralization and osteogenic markers expression in vitro in a calvarial bone defect model in rats; the photoactivated rhBMP4 constructs showed improved bone formation and maturation [354]. In another study, DPSCs were seeded on a 0.3% agarose gel layer with osteogenic media and treated with LLLI that enhanced the osteogenic differentiation as seen by increased mineralization and ALP activity [355].

Stem cells as osteoblast progenitors are essential for bone restoration, and with their osteogenic potential, DSCs are a valuable choice for tissue regeneration—especially when the osteogenesis of DSCs can be improved with the stimulation or inhibition of certain growth factors. The same can be said for the environmental stimulators, which have the potential to improve the osteogenesis of DSCs. However, both growth factors and environmental stimulators could hinder osteogenesis if not used in the right settings. Thus, research focusing on finding optimal concentration and treatment conditions will further ease the transition into clinical applications.

Another key process in bone restoration is angiogenesis, which is responsible for providing the tissues with oxygen and nutrients though the blood supply. In the next section, the angiogenic potential of DSCs will be reported. Specifically, the effect of growth factors and environmental stimulators on improving angiogenesis in DSCs will also be investigated.

Similar to osteogenesis, angiogenesis can be positively influenced using external factors mimicking the microenvironment of the stem cells undergoing differentiation toward endothelial cells and thus starting the process of blood vessel formation.

## 4. Regulators and Enhancers of Angiogenesis in Dental Stem Cells

Angiogenesis is a vital part for bone formation and repair; in particular, the cellular interaction between osteoblasts and endothelial cells (ECs), which are the key players, must be considered [356,357]. Many tissue engineering strategies aim to induce vascularization in grafts, and one strategy to do so is to use stem cells and stem cells-derived ECs [358,359,360,361,362]. DSCs possess pro-angiogenic potential, either by direct differentiation toward ECs or through their paracrine effects [90,363,364,365,366,367].

The angiogenic potential of DPSCs and SHED has been described by different authors [31,32,74,363,368,369,370,371,372]. The work done by Huang and colleagues showed that DPSCs have higher expression and secretion of VEGF than A-MSCs [74]. In another study where DPSCs were differentiated toward ECs, they were described to be comparable to primary ECs. In addition, when EC-differentiated and naive DPSCs were co-cultured in a vascular network formation assay, the naive DPSCs co-localized adjacent to the tubules formed by the differentiated cells in a matter similar to pericytes [372]. Zhang and colleagues showed that when DPSCs and SHED were induced to differentiate toward ECs, they formed de novo blood vessels through a process similar to vasculogenesis, which is observed in early embryonic development [370]. In a study where the endothelial differentiation capacity of SHED and DPSCs was investigated, SHED-derived ECs were shown to have a gene profile more similar to primary ECs [369]. SHED had higher expression levels of most members of the erythroblast transformation-specific family, which are involved in the regulation of angiogenesis and endothelial differentiation [368]. Dental stem cells from carious tissue seem to possess higher proliferative abilities and a different proteomic profile compared to stem cells from healthy tissues [31,32,373,374]. Recently, in a study where CDPSCs and healthy DPSCs were compared, CDPSCs depicted higher expression of VEGF, among other angiogenesis-related factors, including SDF-1 and PDGF [375]. Moreover, Chen and colleagues showed that when cultured in angiogenic induction medium, CDPSCs expressed higher levels of VEGF, PDGF, and SDF-1 [375]. In a tube formation assay, the induced CDPSCs were also able to form vascular-like networks. Quantification of the assay showed that CDPSCs had an enhanced in vitro angiogenesis potential [375]. To conclude, dental stem cells could be utilized in tissue engineering to improve vascularization. In the next sections, the growth factors and environmental stimulators that modulate the angiogenic potential of DSCs will be elaborated.

### 4.1. Growth Factors Regulating Angiogenesis of Dental Stem Cells

Similar to osteogenesis, growth factors affect angiogenesis of DSCs as well. Endothelial cells and vascular smooth muscle cells (SMC) are important building blocks of new blood vessels [376], and DSCs were shown to be able to differentiate toward both subtypes [377]. VEGF is vital for the differentiation of DSCs toward the endothelial lineage, and thus, rhVEGF is a component of the endothelial differentiation supplements. Wu and colleagues investigated the effect of VEGF and IGF-1 on the angiogenic differentiation of CDPSCs [266]. The combination promoted the proliferation and the angiogenic differentiation as seen in tube formation assays. They also observed an activation of the AKT signaling pathway after treatment with either VEGF or IGF-1 or the combination where it was further increased [266]. Recently, it was shown that DPSCs transfected with SDF-1α (DPSCs/SDF-1α) and VEGF (DPSCs/VEGF) exhibited increased proliferation and angiogenesis [378]. Wild-type DPSCs as well as SDF-1α transfected DPSCs stabilized tubes formed by human umbilical vein endothelial cells (HUVECs) in vitro. The injection of a mixture of DPSCs/VEGF and DPSCs/SDF-1α into tooth root canals in a mouse model resulted in significantly increased length of regenerated pulp-like tissue in comparison to that of wild-type DPSCs, DPSCs/VEGF, or DPSCs/SDF-1α [378]. Gorin and colleagues investigated the role of priming bFGF for angiogenesis of SHED. The priming of SHED with bFGF resulted in an upregulation of VEGF and hepatocyte growth factor (HGF) [379]. It also improved angiogenesis, as indicated by the formation of larger vessels when transplanted in a mouse model [379].

The inhibition of TGF-β1 by SB-431542 improved rhVEGF-induced endothelial differentiation of SHED. This effect was due to the inhibition of phosphorylation of Smad 2/3 under stimulation of VEGFA, which upregulated VEGFR2 phosphorylation [370]. On the other hand, TGF-β1 was shown to induce SHED into smooth muscle cells [377]. The differentiated cells expressed smooth muscle markers such as Calponin, SM22α, and α-SMA. Moreover, co-culture with HUVECs on Matrigel showed that SHED-derived SMCs enhanced vascular formation, and the effect was better than for primary SMCs alone [377]. The treatment of SHED with BMP4 could not induce the cells to SMCs, and the combination of TGF- β1 and BMP4 resulted in a weaker differentiation in comparison to TGF-β1-treated cells, possibly because of the competition of BMP4 with TGF-β1 for binding to TGF-β receptors (TGF-β RI and RII). Using a TGF-β1 inhibitor, they could demonstrate that TGF-β1 induction is most likely mediated by the TGF-β1-ALK5 signaling pathway [377]. Recently, DPSCs were also induced to SMCs through treatment with TGF-β1 and PDGF-BB, in combination with pre-coating with gelatin, which could slightly enhance the differentiation [380].

The modulation of angiogenesis and osteogenesis by growth factors could be very useful for bone regeneration. However, there are some issues that need to be addressed—for instance, which growth factor/combination of growth factors will support bone regeneration and vascularization. It is also important to consider the time point during the differentiation at which the cells are treated with the growth factors and the duration of treatment. In Section 8, advances on delivery of growth factors and drug release from scaffolds will be discussed.

### 4.2. Environmental Stimulators Regulating Angiogenesis of Dental Stem Cells

Under physiological conditions, hypoxia results because of ischemia, and/or blood vessel damage upregulating the expression of pro-angiogenic growth factors, such as VEGF or HIF-1 [381,382]. HIF-1 is an oxygen-sensitive transcriptional activator that is considered a vital mediator of the response to hypoxic conditions. In line with this, the HIF-1 pathway was found to be a master regulator of angiogenesis through its regulation of pro-angiogenic factors and cytokines either directly or indirectly [383]. Angiogenesis initiated by HIF-1 is mostly mediated through VEGF [384,385]. When cultured in hypoxic conditions, DPSCs depicted an increased expression of HIF-1α accompanied by an upregulation of VEGF expression. In addition, HIF-1α inhibition partially inhibited VEGF expression [386]. Culturing SCAP in hypoxic conditions increased their secretion of VEGFA in vitro [387]. When SCAP and HUVECs were co-cultured in hypoxic conditions, the enhanced expression of angiogenesis-related proteins such as HIF-1 and VEGF and the release of VEGF was observed [388]. Sun and colleagues demonstrated that hypoxia facilitated and enhanced the expressions of HIF-1α and small ubiquitin-like modifier-specific protease 1 (SENP1) in DPSCs and that both have a positive feedback loop during angiogenesis [389]. In addition, Bcl-2 overexpression enhanced angiogenesis through HIF-1-mediated VEGF secretion in tumors [390,391]. Recently, Zhang and colleagues demonstrated that Bcl-2 overexpression could improve the in vivo post-implantation cell viability and the VEGF-mediated angiogenic and vasculogenic differentiation of DPSCs [392]. This was evident when Bcl-2 transduced DPSCs demonstrated increased VEGF expression under hypoxic conditions. They also found that HIF-1 transcription might not be the only factor responsible for the increased VEGF expression in Bcl-2 transduced DPSCs under hypoxia, which is evident by the increased VEGF and absent HIF-1 expression in normoxia [392]. Thus, the precise mechanism by which Bcl2 increases VEGF expression remains to be elucidated. A better understanding of the effect of hypoxia on DSCs will be beneficial in optimizing graft, as cells in such constructs are usually in hypoxic conditions, especially if the hypoxic conditions could improve vascularization inside the graft, which is considered one of the major challenges of tissue grafts for transplantation [393].

However, the differentiation process of the dental stem cells toward osteoblast or endothelial cells is far from the generation of three-dimensional bone tissues. The first steps toward such tissues are discussed in the next section.

## 5. Hard Tissue Regeneration Approached by Dental Stem Cells

In this section, the advances made with dental derived stem cells using cell sheets and 3D spheroids will be discussed. Cell sheets technology and spheroids are being studied for their potential in bone regeneration as a scaffold-free strategy. Both techniques have an advantage to cell suspensions/injections, which can be limiting, since it is hard to localize the cells or control the tissue shape when injecting, not to mention the limited quantity that can be injected and the cell loss that occur after injection due to the procedure [394]. Cell sheets are generated by culturing the cells on a thermoresponsive polymer surface, which allows harvesting of the cells with a non-proteolytic enzymatic method. This allows the preservation of the cell–cell junction and the extracellular matrix. Cell sheets are flexible and can be shaped according to the needs, allowing a uniform distribution of cells resulting in an approach, which is one step closer to tissues in vivo [395,396,397,398,399].

Cell sheets were generated using DPSCs [400,401,402,403] and PDLSCs [95]. Cell sheets generated from DPSCs were shown to be able to retain their undifferentiated state and osteogenic capacity [400]. DPSCs were able to form 3D structure resembling woven fibrous bone after 40 days of in vitro culture, which is a structure that could be used for bone regeneration [404]. When implanted dorsally in vivo, the woven bone constructs became a highly vascularized remodeled bone tissue. When implanted into a rat model of a mandibular bone defect, the constructs formed mature bone of the lamellar type and structures resembling Haversian canals [404]. Stem cell sheets were also tested for cleft palate repair [403]. Cell sheets were made from either BM-MSCs or SHED and were able to mineralize in vitro. SHED cell sheets expressed the osteogenic markers (OSX, OCN, and OPN) after implantation in ex vivo-cultured embryonic palatal shelves and in ovo culture. When the sheets were cultured in a chorioallantoic membrane model, BM-MSC cell sheets displayed a single layer morphology, and there was a strong localization of the premature and mature osteoblastic markers (OSX and OCN) in the cell sheets. Meanwhile, SHED cell sheets formed multilayered structures, showing weak OSX localization and a strong OCN and OPN localization in the cell sheets. Mineralization and gene expression studies indicated that SHED cell sheets had more potential to regenerate bone than BM-MSC cell sheets [403]. Tanaka and colleagues attempted vascularized bone tissue formation in vitro utilizing cell sheets and ECs and erythrocytes-based beads [401]. They used DPSCs induced toward osteoblasts and ECs from the dental pulp and prepared a compact layer (made of osteoblast sheets and EC and erythrocytes-based beads) and spongy layers (composed of osteoblast-based beads and ECs and erythrocytes-based beads), which were then organized in a 3D tissue construct and cultured for 30 days in vitro. The use of erythrocytes and ECs in the beads was meant to increase oxygen delivery and vessel capacity and to mimic the formation of blood vessel during embryogenesis. The construct showed the structural differences between compact and spongy bone, and histological analyses showed bone lacunae-containing osteocytes, Haversian canal-like structures, and extensive vascularization. They also detected the presence of TRAP-positive osteoclast-like cells, although the origin of these cells still needs to be elucidated [400]. Taken together, they successfully generated bone tissue that closely resembled native tissue, possibly possessing bone remodeling ability.

In another study, the enhancement of DPSCs cell sheets was tested using the combination of the commonly used medium supplemented with vitamin C and photobiomodulation [402]. The combination of Vitamin C and PBM, in comparison to vitamin C alone, enhanced the formation of CSs and the osteogenic function of the cells. Moreover, PBM preserved cellular longevity, as shown by telomerase activityand gene expression of OCT4 and Mitofilin [402]. Three-dimensional (3D) cell sheets were also constructed using a mixture of PDLSCs and HUVECs, and their osteogenic and angiogenic abilities were examined [95]. The results showed that co-culture cell sheets possess high levels of osteo and odontogenic markers with signs of initial vascular formation. Specifically, higher ALP expression and mineralization were observed in mixtures of PDLSCs–HUVEC after 3 days of culture. The co-culture constructs showed higher expression of BSP and RUNX2 at 7 days. With regard to angiogenesis, histological staining of the constructs showed layered cell sheet structure with endothelial cell islands [95].

Cell sheets could also be used for purposes of tooth replacement and regeneration. Monteiro and colleagues attempted creating a biomimetic 3D tooth bud model consisting of dental epithelial (DE) and dental mesenchymal (DM) cell sheets [405]. The cells were isolated from tooth germs and CSs were generated. Then, the CSs were combined with biomimetic enamel organ and pulp organ layers produced using hydrogels to create a biomimetic 3D tooth. In Vitro, the constructs expressed the odontogenic markers BMP2, RUNX2, tenascin, and syndecan. When the 3D tooth bud constructs were implanted subcutaneously in rats, they were able to form mineralized tissue expressing BMP2 and RUNX2 [405]. Park and colleagues designed PDLSCs sheets as a model for tooth root and periodontal tissue regeneration [406]. The PDLSCs sheets were osteogenically induced in vitro pretreated with BMP2 and then grafted on micro/macroporous biphasic calcium phosphate (MBCP) blocks. When transplanted subcutaneously in mice, the BMP2 pretreated constructs exhibited higher mineralization and collagen deposition compared to the untreated group [406].

Spheroids are multicellular spherical clusters formed by 3D self-aggregation [407]. Three-dimensional (3D) spheroids were shown to have effects on the ECM and growth factors production of stem cells [408,409,410]. When cultured as 3D spheroids, DFSCs had an enhanced osteogenic potential in vitro [411]. Sano and colleagues investigated the potential of co-cultured spheroids of PDLSCs and HUVECs for periodontal tissue regeneration in vitro and in vivo [412]. When PLDSCs and HUVECs spheroids were cultured in a ratio of 1:2, the expression of OCT4 and NANOG was increased. The spheroids also had increased levels of VEGF. When induced toward osteogenesis, the co-culture spheroids showed higher expression of osteogenic markers in comparison to a monolayer culture or spheroids with PDLSCs only. Moreover, PLDSCs and HUVECs spheroids with ratios of 1:1 and 2:1 regenerated more bone volume in a periodontal tissue defect model in rats [412]. Na and colleagues introduced a protocol for the formation of 3D SCAP-derived stem-cell sheet-derived pellets (3D SCAP-CSDP) and tested them for their odonto and osteogenic capacity for tooth regeneration [413]. They generated cell sheets that were formed into pellets. The pellets had a higher gene expression of ALP, DSPP, BSP, and RUNX2 mRNA in comparison to the CSs alone. When the pellets were inserted into human treated dentin matrix fragments and implanted in a mouse model, the CSDPs filled the root space with dental pulp-like tissue. The tissue showed good vascularity and contained a continuous layer of dentin-like tissue, which was deposited onto the existing dentin. Moreover, the surface of this newly formed dentin contained odonto/osteoblast cells that expressed osteogenic markers [413].

Ultimately, cell sheets and 3D spheroids are promising tools for hard tissue regeneration. It has been shown that it is possible to generate such a construct with DSCs. Co-culture techniques allow the generation of complex multipurpose constructs. These have potential in applications for repair and replacement strategies for bone and tooth defects. However, cell-free therapy approaches have been considered also and are discussed in the next chapter.

## 6. Cell-Free Therapy Approaches Using the Dental Stem Cell-Derived Secretome

The therapeutic effect of MSCs in tissue repair is not limited to their ability to differentiate. The paracrine activities due to their secretome components are also an important factor. The MSC secretome include soluble proteins such as cytokines and growth factors and extracellular vesicles such as exosomes and microvesicles, which carry proteins, miRNAs, and mRNAs [414,415]. In Vitro and in vivo preclinical studies showed promising results for using the MSC secretome in bone regeneration [416,417,418,419]. In Vitro, the secretome of MSCs can be found in the medium the cells are grown in, which is branded as “conditioned media”. The advantage of using cell-free therapy approach is that it might be safer than transplanting proliferating cells, which might differentiate toward unwanted lineages and have the risk of activating an allogeneic immune response. Thus, with clinical applications in mind, it is easier to evaluate the safety and potency of the secretome, and it is more economical and effective with regard to storage and mass production [420,421,422]. Here, we will summarize the data from recent studies on using the DSCs secretome as a tool for hard tissue regeneration.

DPSCs-derived conditioned medium (DPSCs-CM) was able to improve the angiogenesis of HUVECs in vitro [423]. Regardless of the concentration of CM used, adhesion, proliferation, migration, and tube formation in HUVECs were enhanced. This enhancement could be a result of the expression of anti-apoptotic markers and mitogens (TIMP-1, VEGF and uPA) within the CM. SHED-derived conditioned medium (SHED-CM) was also shown to enhance the proliferation and migration of HUVECs in vitro and in vivo [424]. SHED-CM was investigated as an approach for the reconstruction of the alveolar clefts palate in a mouse model [425], and SHED-CM was more effective in regenerating bone defects compared to SHED, with better osteogenesis and vascularization in the defects. Analysis of SHED-CM showed that it contained an abundance of the osteogenic markers (OPG, OPN, BMP2, and BMP4) and angiogenic markers (M-CSF, MCP-1, ANG, bFGF, VEGF-C, and VEGF-A) [425]. Another group even investigated loading titanium implants with SHED-CM, which enhanced BM-MSCs attachment to the implants and showed promising results for bone formation in a canine femur bone defect [426]. The CM derived from SCAP had a diverse secretome profile, with proteins involved in angiogenesis, immunomodulation, or those with anti-apoptotic abilities [427]. The expression profile and angiogenic potential of SCAP-CM was affected by culture and environmental conditions [90]. SCAP-CM isolated from SCAP cultured in serum-deprived conditions with low glucose as well as hypoxic conditions showed a better induction of vasculogenesis in HUVECs compared to CM from SCAP grown under normal conditions. Comparing SCAP-CM and BM-MSCs-CM showed that SCAP-CM possessed potential to enhance the proliferation and osteogenic differentiation of dental pulp cells [428].

The potential of exosomes isolated from osteogenically differentiating DPSCs in inducing odonto and osteogenic differentiation of naïve DPSCs and MSCs was assessed [429]. The DPSCs from which exosomes were isolated were treated either with growth medium (DPSC-Exo) or with osteogenic differentiation medium (DPSCs-OD-Exo). Both cell types were able to uptake the exosomes. In DPSCs, the uptake was mediated by the p38 MAPK pathway, which in turn upregulated the gene expression of BMP2 and BMP9. Moreover, the gene expression of some growth factors and osteogenic markers (RUNX2, COL1, OPN, and DSPP) were upregulated in DPSCs after they were treated with exosomes from both CM types. In a 3D culture model in vitro where exosomes were loaded in collagen hydrogels, they increased the osteo and odontogenic differentiation of DPSCs. In addition, the increase was more robust in comparison to 2D culture. Additionally, the DPSC-OD-Exo triggered a stronger increase in the expression of odontogenic and osteogenic marker genes compared to DPSC-Exo. In MSCs cultured in the 3D in vitro model, treatment with DPSC-Exo and DPSC-OD-Exo increased the expression of several growth factors and ECM proteins along with RUNX2 and DSPP. This indicates the potential of osteo/odontogenic induction of stem cells from other tissues and via exosomes of different origins [429]. Zhuang and colleagues showed similar results, with SCAP-derived exosomes increasing the osteogenic potential of BM-MSCs in vitro and in vivo [88]. In Vitro, their results showed that increasing concentrations of exosomes effectively increased mineralization and DSPP expression in BM-MSCs [88].

The role of microRNAs contained in DPSCs-derived exosomes and their potential signaling cascade in osteogenic and odontogenic differentiation was assessed [430]. Similar to the studies described before, the exosomes were isolated from undifferentiated (DPSC-Exo) and osteogenically differentiated DPSCs (DPSCs-OD-Exo). Treatment with DPSCs-OD-Exo had better potential to induce the osteo/odonto differentiation of naive DPSCs, as shown by the increased expression of specific markers (DSP, DMP-1, ALP, and RUNX2). In comparison, DPSCs-OD-Exo showed 28 significantly different microRNAs, of which seven were increased and 21 decreased. The TGF-β pathway was targeted by the microRNAs, with 16 genes targeted by 15 differentially, expressed microRNAs. DPSCs-OD-Exo-treated DPSCs had an increased expression of TGF-β1, TGF-R1, p-Smad2/3, and Smad4. Moreover, the inhibition of the TGF-β1 pathway decreased the protein expression of p-Smad2/3, DSP, and DMP-1 [430]. DPSC-Exo loaded in a fibrin gel scaffold was able to enhance the proliferation and migration of HUVECs in a monolayer and 3D culture system [431]. More importantly, co-cultures of HUVECs and DPSCs treated with DPSC-Exo showed improved vascularization and an increased release of VEGF.

As DPSCs from diseased teeth were shown to have better proliferative and osteogenic abilities [31,32,432], Zhou and colleagues used exosomes from DPSCs isolated from periodontally compromised teeth (P-DPSC-Exo) to enhance angiogenesis [433]. HUVECs were treated with P-DPSC-Exo and exosomes from healthy control DPSCs. Both exosome types were able to improve angiogenesis markers expression, proliferation, migration, and tube formation in vitro. P-DPSC-Exo was shown to be more effective in enhancing vascularization. In a mouse model with a skin defect, both treatments with either of the exosomes resulted in faster wound healing and improved vascularization, with P-DPSC-Exo preforming better.

Wei and colleagues investigated SHED-derived exosomes (SHED-Exo) for recovery after bone loss caused by periodontitis [434]. Treatment with SHED-Exo significantly restored bone tissue loss caused by periodontitis in vivo. Additionally, they examined the effect of SHED-Exo and SHED-CM on cell viability and osteogenesis of BM-MSCs in vitro. Both SHED-Exo and SHED-CM were able to reduce cell apoptosis and enhance proliferation, with a higher effect in the latter. Both SHED-CM and SHED-Exo increased the osteogenic markers expression and mineralization of BM-MSCs, with SHED-Exo showing slightly better effects. Wang and colleagues studied the effect of SHED-derived exosomes (SHED-Exo) on the osteogenesis of PDLSCs in vitro [435]. Exosomes were derived from undifferentiated SHED and SHED, which were osteogenically differentiated. The treatment of undifferentiated PDLSCs resulted in increased cell viability with an increasing concentration of exosomes. More importantly, SHED-Exo treatment showed enhanced osteogenesis in vitro, which was more prominent in differentiating PDLSCs treated with osteogenic induction medium and the SHED-Exo in comparison to PDLSCs, which are induced with osteogenic medium alone, or PDLSCs treated with SHED-Exo only. SHED-Exo treatment led to enhanced mineralization, ALP activity and osteogenic gene markers expression (RUNX2, OPN and OCN). This effect of SHED-Exo was mediated by BMP/Smad signaling and Wnt/β-catenin signaling in PDLSCs, where Smad1/5/8 phosphorylation was enhanced, and nuclear β-catenin protein increased expression. Moreover, increased levels of Wnt3a and BMP2 were observed in SHED-Exo, which was shown to be important in enhancing the osteogenesis of PDLSCs.

Conditioned medium and exosomes isolated from dental stem cells were shown to improve angiogenesis and osteogenesis. This can be interesting for regeneration, especially considering the lower risks of a cell-free approach, which is in line with an easier approval procedure, especially considering European laws. Cell-free approaches might be beneficial for small defects, whereas critical size bone defects benefit from larger tissue replacements, which are strategies that rely on scaffolds mimic the tissue and improve cell fate within. These strategies will be discussed in the next chapter.

## 7. Stem Cell Therapy Approaches with Scaffolds

This section will briefly discuss stem cell-based therapy approaches, which combine the use of stem cells and advanced biomaterials, in particular scaffolds and release materials for hard tissue regeneration. Three-dimensional (3D) scaffolds are essentially required to support three-dimensional stem cell proliferation and final 3D tissue (bone) generation. Today, research groups worldwide try to adapt the scaffolds in size, porosity, and surface characteristics (e.g., number and nature of functional groups) to the specific cell type and clinical application [436].

Furthermore, the stem cell fate is influenced by mechanical stimuli of different origin and strength (e.g., mechanical tension as tensile strain, mechanical stretch, mechanical loading, micro- and nano-scale surface topographies, and others). In a recently published mini-review, Marelli and colleagues summarized and discussed original data describing the regulating effects of mechanical stimuli on the differentiation behavior of DSCs [437]. Therefore, future tissue engineering approaches for teeth and bone generation have to consider the sensitivity of DSCs toward different mechanical stimuli. Moreover, the underlying molecular mechanisms have to be studied. The interaction between stem cells in general and dental stem cells with scaffold biomaterials have been studied on different levels including the superficial macroscopic level and the structural microscopic level. In the case of superficial macroscopic interactions, a significant influence of geometrical and mechanical properties of the scaffold materials on cell response to mechanical stimulations have been confirmed [438].

**Figure 5:**
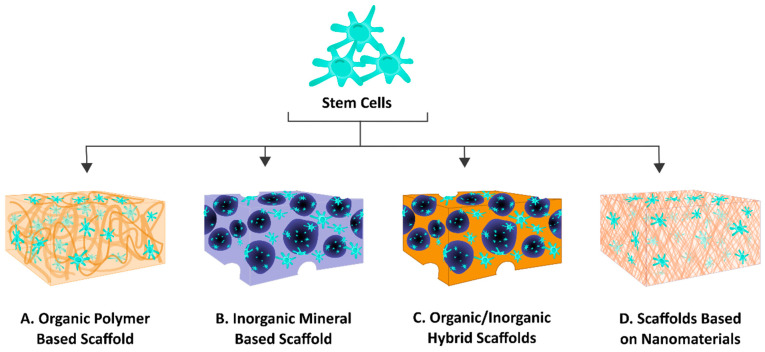
Scaffold types used for stem cell-based tissue engineering approaches: (**A**) Organic polymer-based scaffolds; (**B**) Inorganic mineral-based scaffolds; (**C**) Organic/inorganic hybrid scaffolds consisting of polymer and mineral components; (**D**) Scaffolds based on nanomaterials.

Due to their chemical composition, current scaffolds used for stem cell-based hard tissue engineering could be divided into the following groups: polymer-based scaffolds, mineral-based scaffolds, hybrid scaffolds composed of mineral and polymer components, and scaffolds based on nanomaterials (Figure 5). For hard tissue engineering applications, hybrid scaffolds are the favored materials due to their similarity with natural bone.

Since the focus of this review article is mainly directed toward biological aspects, only a very few examples will briefly be discussed here. For further information, a recently published review covers details on scaffold development with special focus on bone physiological microenvironment and healing mechanisms [439].

A few of the most interesting scaffold examples used in the context of dental stem cells should be briefly discussed. Recently, Jafar and colleagues reported the development of dentin-derived proteins [440]. Dentin was shown to be not able to induce the endothelial differentiation of DPSCs even in the presence of angiogenic factors; a combination of DPSCs and scaffolds loaded with growth factors is required for the successful regeneration of periodontal defects [440,441]. The human dental pulp contains a slight subpopulation of stem cells that exhibit multipotency and thus the ability to differentiate into odontoblasts, neural cells, and vascular endothelial cells. Thus, DPSCs are uniquely suited for dental pulp tissue-engineering purposes. Sakai and colleagues reported a tooth slice/scaffold model: a powerful tool for mechanistic studies designed to understand the processes of DPSCs differentiation [442].

The most common diseases linked with teeth and their supporting tissues are periodontal disease, caries, and traumatic injuries. External interventions are often necessary to promote the biological repair of damaged dental tissue. The concepts in restoring damaged tissues have undergone significant change, from substitution to restoration or replacement, and finally regeneration. Since stem cell-based tissue engineering and regenerative medicine emerged two decades ago, these novel therapeutic strategies have been evaluated for their potential to replace, repair, maintain, and enhance tissue or organ function [443]. The strategy encompasses numerous elements, including biomaterials, stem cells, tissue-inducing substances, or biomimetic regenerative environments [444].

Bone loss is a major result of periodontitis. Pathogenic microorganisms in the biofilm, environmental issues such as tobacco use, and genetic factors can all contribute to periodontitis and bone loss. Losing the supporting bone around a tooth results in tooth movement and dislocation, eventually leading to tooth loss. Various techniques have been developed to support and enhance the osteogenesis process. New bone and new periodontal connective tissue attachments could be obtained [445]. Clinical trials showed that periodontal bone filling was observed. Here, xenografts have been used—for example, Bio-Oss^®^ [444]. One study investigated the effects of employing titanium mesh in conjunction with Bio-Oss^®^ for localized alveolar ridge augmentation [446].

For dental pulp regeneration, there are primarily two approaches: (a) pulp revascularization and (b) scaffold and/or stem cell-based pulp regeneration. Dental pulp revascularization usually depends on inducing host cells from the apical region to migrate into the root canal and differentiate into a vascularized pulp tissue. Wei and colleagues successfully transplanted a root shape HA/TCP scaffold containing allogeneic dental pulp stem cells covered by a vitamin C-induced allogeneic periodontal ligament stem cell sheet into the jawbones of miniature pigs [447].

A variety of studies confirmed the osteoconductive capacity and the ability of nanofibrous scaffolds to support the growth of clinically relevant bone tissue-engineered bio-implants [448]. The morphological features of electrospun nanofibrous scaffolds are designed to mimic the ECM of natural tissue, and those scaffolds possess a wide range of unique physical and biochemical properties [449]. They enable cell adhesion, proliferation, migration, differentiation, and improve cell lifespan, which is needed for cell-based therapy. Based on the structure and mechanics of the target tissue, the desired scaffold can be based on natural or artificial materials of varying mechanical strength [450]. Recent studies reported by the groups of Diomede and Trubiani, using human oral stem cells and PLA scaffolds, confirmed the therapeutic potential of scaffold-based approaches for the reconstruction of bone tissue defects [451,452].

Both natural and synthetic polymer scaffolds provide distinct advantages regarding cell attachment, proliferation, and angiogenesis. The stem cell differentiation and resulting biochemistry of tissue repair strongly depend on the chemical structure of the polymers and their 3D architecture [453]. Promising natural materials used for scaffold synthesis are collagen, fibrin, chitosan, hyaluronic acid, alginate, and peptides. In the case of synthetic polymers, polylactic acid (PLA), polyglycolic acid (PGA), and corresponding copolymers were successfully tested in vitro and in vivo [122,453]. Bakopoulou and colleagues reported a study using DPSCs embedded in chitosan/gelatin scaffolds for enhanced orofacial bone regeneration in customized constructs [454]. Today, hybrid systems, combinations of mineral and polymer materials, are the most popular and promising materials. Forni and colleagues designed highly porous 3D biodegradable scaffolds consisting of inorganic CaSi/dicalcium phosphate dihydrate (DCPD) doped with poly (α-hydroxy) acids (such as PLA) used for stem cell-based bone regeneration. Results showed vascular remodeling due to the strong angiogenic attitude of the hybrid scaffolds [455]. Tatullo and colleagues investigated mineral-doped hybrid scaffolds, composed of PLA, DCPD, and/or hydraulic calcium silicate [122]. Microcomputer tomography (CT) revealed an interconnected highly porous structure for all hybrid systems with up to 99% open pores. These scaffolds were colonized with autologous stem cells from a periapical cyst, showing promising results in the regeneration of periapical and alveolar bone [122]. Oryan and colleagues seeded stem cells onto tissue-engineered osteoinductive hybrid scaffolds consisting of gelatin, nano-HA, and bioactive glass. Results showed enhanced healing of critical-sized radial bone defects in rats [441]. Scaffolds were tested and characterized by X-ray diffraction (XRD) and scanning electron microscopy (SEM) and analyzed for porosity and degradation rate. Obviously, the combination of osteoconductive HA with bioactive glass components could effectively accelerate the bone regeneration process.

## 8. Drug Release Concepts, Mechanisms, and Applications in Stem Cell-Based Osteogenesis and Angiogenesis

The previous sections have elaborated that osteogenesis and angiogenesis are extraordinarily complex processes involving a well-orchestrated interplay of various cell types and signaling molecules. The regeneration of defects can be significantly supported by using biomaterials. In addition to the mechanical and structural support of the defect, scaffolds represent a framework for involved cells and the tissue to be formed. Therefore, scaffold materials with e.g., proliferation-promoting properties outperform inert materials. In order to further increase the biological effectiveness of the scaffold biomaterial, it can be loaded with osteo- and angiogenesis-promoting agents. With this approach of local drug delivery, the therapeutic efficacy can be focused to the defect site, yielding high local concentrations and low systemic concentrations that lead to a reduction of adverse side effects. The effectiveness of drug delivery systems can be further improved by time-controlled drug release to ensure a constant drug supply over several weeks of defect regeneration. In the previous sections, it was also shown that different drugs and drug concentrations, respectively, are particularly beneficial, depending on the stage of tissue regeneration. Thus, release materials possessing multiple release mechanisms or materials that release the active ingredient in response to a certain stimulus are the focus of recent research [456,457,458,459,460,461].

As shown and discussed in Section 3 and Section 4, dental stem cells are very sensitive to a huge number of different criteria such as growth factors, other signaling molecules, pH value, oxygen concentration, mechanical stress, etc. (see Table 1). Moreover, environmental stimulators are regulating osteogenesis in dental stem cells. Therefore, with the help of drug release, it is possible to guide and trigger the differentiation process of dental stem cells into specific cells (e.g., osteoblasts). In the case of critical size defects, angiogenesis is a key process for successful tissue growth, providing oxygen and nutrients via blood supply. Studies confirmed that both the osteogenic and angiogenic potential of stem cells is affected by growth factors, which could be provided by tailored release materials [439]. The following section gives an overview of general concepts and mechanisms of drug delivery, the materials and manufacturing methods used, as well as some recently published examples for stem cell-based therapies. In many cases, a drug release material simultaneously acts as a scaffold material for the defect site. In this case, the material has to meet the same criteria as sole scaffold materials such as biocompatibility, biomimicry, sufficient mechanical properties and porosity for cell migration and nutrient supply, and biodegradability without the formation of toxic metabolites [462]. Analogue to scaffolds, a vast majority of drug release materials consist of polymers, inorganic compounds, or hybrid composite materials. Polymers used for drug delivery purposes may be divided into synthetic compounds (such as poly(ethylene glycol), poly(ε-caprolactone), poly(lactic acid), or poly(lactic-co-glycolic acid)) and natural polymers such as peptides (e.g., gelatin, collagen) or polysaccharides (e.g., alginate, chitosan, hyaluronic acid) [462,463,464]. Porous polymeric materials are designed to resemble the highly hydrated extracellular matrix and provide sufficient nutrition to the defect site. However, they often lack mechanical stability and do not provide favorable biological properties by being bioinert. In contrast, inorganic materials such as calcium phosphates [465,466], calcium silicates [467,468], mesoporous silica [469], or bioactive glasses [470] are often rigid structures with sufficient mechanical and biological properties toward cells but lack proper nutrient supply to the defect site. Hybrid composite materials combine the favorable properties of the single materials by adding bioactive properties and the mechanical stability of inorganic materials to the porous structure of polymeric materials [471].

Drug release materials usually appear in the form of hydrogels [472,473] or fibrous textures [474] in case of polymers and granules, ceramics, or cements in case of inorganic materials [461,475]. While the shape and porosity of fibrous textures is mainly determined by a proper manufacturing method such as electrospinning, there are multiple ways to achieve desired shapes and porosities for both polymeric hydrogels and inorganic materials such as salt leaching, freeze drying, gas foaming, and 3D printing. Additionally, sintering processes are established for ceramic and cementitious inorganic scaffolds [463,464,476].

In addition to the simultaneous use of scaffold materials as drug release materials, microparticles, microspheres, nanotubes, nanofibers, or liposomes and micelles may be used as drug release systems alone or in combination with scaffolds of different composition [477]. Loading a delivery system with drugs or signaling molecules can be achieved by either covalently linking the drug and carrier or by using physical interactions between the drug and the material [464,478]. For the latter, it is possible to simply add the drug during the manufacturing process of the material or perform the drug loading after the manufacturing process by either soaking the material in a solution of the drug or by injecting a defined amount of drug solution into the material, preparing a physical blend [462,472].

**Figure 6:**
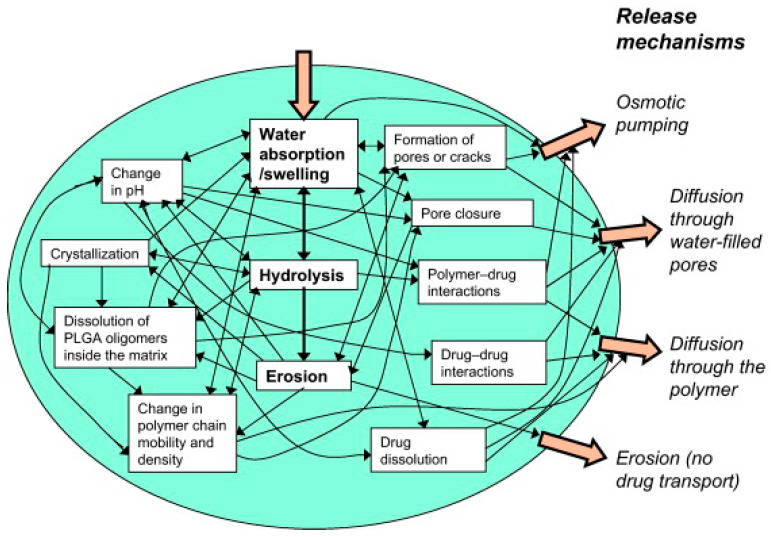
The complex picture of the different factors that influence drug release from PLGA matrices. Arrows illustrate the effects of the properties of the DDS and the surrounding environment on the processes that, in turn, influence drug release. Reprinted with permission from Fredenberg et al. (2011) [479]. Copyright 2021 Elsevier.

The mechanisms and thus kinetics of drug release from delivery systems are extraordinarily complex processes influenced by multiple interdependent parameters. This complexity is exemplarily illustrated by Fredenberg and colleagues for the correlation of various parameters in PLGA-based drug delivery systems [479]. As Figure 6 shows, properties of the raw material used, properties of the manufactured drug delivery system, properties of the encapsulated drugs, and influences of the release environment can be considered as isolated input parameters. When the single components are combined to a drug delivery system, input parameters are merged and affect each other to develop the drug delivery system’s properties, which then ultimately determine the mechanisms of drug release such as osmotic pumping, diffusion through water-filled pores, diffusion through the polymer, and erosion of the material (to specify the most important ones).

When discussing drug release systems that are not PLGA-based as in this example, some of the input parameters may change, but the immense complexity and interdependency of properties and criteria to be recognized remains and displays one of the main challenges in the field of controlled drug delivery. Furthermore, the comparability of scientific studies is strongly restricted because variations of single input parameters may simultaneously influence several properties of the delivery system as a whole and thus the drug release kinetics.

Consequently, current research in the field of drug delivery aims at understanding and controlling the structure–property relationships to ultimately establish drug release applications with desired properties, as described in the beginning of this section. Currently, there are just a very few examples reported of drug release studies using dental stem cells, which is most probably due to the complex release kinetics. In Vitro and in vivo studies have recently been reported for a chitosan (CS)-based sponge loaded with TGF-β3 to study the drug influence on the proliferation and differentiation mechanism of primary human periodontal ligament stem cells. Results confirmed that TGF-β3/CS promotes osteogenic differentiation of PDLSCs, which may involve the mitogen-activated protein kinase (MAPK) signaling pathway. The TGF-β3/CS sponge possesses three-dimensional porous interpenetrating pore structures, resulting in a large internal surface area and high porosity (85.65% ± 3.5%). Current tests involve the repair of incomplete alveolar bone defects [480]. Another chitosan-based dual-modular GFs delivery system has been developed by Tang and colleagues to enhance bone regeneration. The first module consists of a porous chitosan scaffold loaded with rhBMP2, while the second one is loaded with VEGF and in situ fixed in the hollowed channels of the first module. In Vitro and in vivo studies revealed differentiated release profiles of the dual system [481].

Two other approaches for a dual delivery of different drugs have been reported by Cheng and colleagues [482] and Aksel and colleagues [268]. In detail, Aksel and colleagues used a gelatin matrix for the delivery of VEGF and BMP2 to study the influence on angiogenic and odontogenic differentiation of DPSCs. Results confirmed higher expressions of angiogenic factors such as PECAM as well as odontogenic factors (e.g., BSP, DMP-1, OCN, and CBFA) compared to unloaded reference systems (*p* < 0.05) [372]. Using a layer-by-layer technique, Cheng and colleagues fabricated electrospun nano fibered core−shell SF/PCL/PVA mats loaded with bone morphogenetic protein 2 (BMP2) (in bulk) and CTGF attached to the surface. Both in vitro and in vivo experiments showed improved MSC osteogenesis [482].

In addition to these rare release studies reported for dental stem cells, there are promising data available for the controlled release of growth factors and signaling molecules in other stem cells (such as mesenchymal), which could in the future be transferred to dental stem cells (see examples in Table 2). Today, highly advanced systems are already tested for various applications including the sequential release of multiple drugs (such as signaling molecules, activators, and suppressors). So-called on-demand release systems are developed using specific stimuli such as pressure, electrical, or photochemical signals [483].

In conclusion, the limited number of reported studies underlines the significance of further intensive efforts in drug release materials development considering all criteria discussed in this chapter. A better understanding of the detailed mechanism is required in order to tailor both the drug loading and release kinetics. Moreover, scaffold and release materials interact and therefore both influence the release kinetics. As various recent studies could confirm, the final 3D microenvironment consisting of appropriate scaffolds and release materials determines the time-controlled drug delivery. In turn, only a precisely adapted construct will guarantee an optimal cascade of cell adhesion, differentiation, proliferation, and growth.

Moreover, future biomaterial development is forced to consider sustainability aspects such as raw material origin, availability, and accessibility. Therefore, research activities are directed toward the exploitation of renewable resources not used as food and feed source as well as biomass waste [485]. A broad variety of crop genotypes is under current investigation, among them so-called low-input crops cultivated in arid conditions [486,487,488,489]. Promising data are reported for lignin-based hydrogels and hybrid materials tested regarding their antimicrobial activity [490]. Very recently, the first lignin-derived micro- and nanoparticles were reportedly used for drug encapsulation and controlled release [491,492,493]. In future, advanced experimental studies will be combined with theoretical modeling [494] as well as multivariate data processing [495] to tailor the biomaterial development and gain a fundamental understanding of the complex release processes. Nevertheless, already now for some few promising approaches, the next step has been done, entering clinical studies, which will be discussed in the next chapter.

## 9. Current Clinical Trials

In recent years, the understanding of dental stem cell biology showed a significant increase based on experiments in vitro and in vivo experiments in animals. In this report, we evaluated the status of the translational efforts using DSCs. In order to evaluate the status of the clinical use, a systematic literature review regarding clinical trials and the clinical trial database was performed. For the literature review, the PubMed database was searched on the 12 December 2020 using the search string: “dental stem Cell” OR “dental epithelial stem cells” OR “dental epithelial stem cells” OR “dental pulp stem cells” OR “stem cells from the apical papilla” using the PubMed Filter “Clinical Trial”. For an outlook on the clinical trial activities, clinical trials on the ClinicalTrials.gov databases were searched on 12 December 2020 using the key term “dental stem cell”. To limit the search on ongoing trials, the following filters “Not yet recruitment” OR, “recruiting, OR “enrolling by invitation”, OR “active not recruiting” were applied. Both database searches and the respective selection of the publication or clinical trials were performed by one author. In the literature review in Pubmed Database, in total, 127 publications were identified. Of these, 16 publications were excluded, since they report no clinical use or were not clinical trials or clinical studies, and 104 reports on clinical studies were excluded, since the clinical trials did not investigate dental stem cells or use dental stem cells as an intervention.

**Figure 7:**
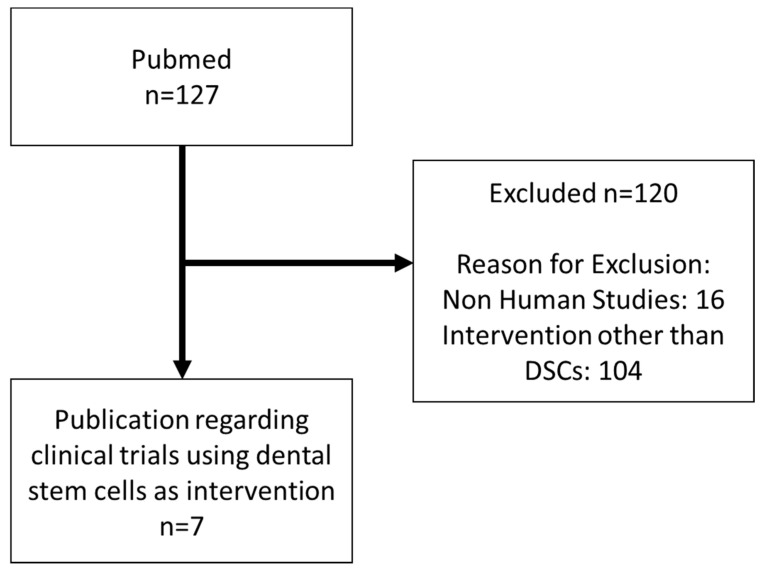
Systematic review flow chart of the systemic literature review and general characteristics of the included publications. In total, 127 publications were identified, of which 16 publications were excluded since they reported no clinical use or were not clinical trials or clinical studies, and 104 reports on clinical studies were excluded, since the clinical trials did not investigate dental stem cells or use dental stem cells as an intervention. A total number of seven publications reported the clinical use of dentals stem cells or used dental stem cells as an intervention.

A total number of seven publication reported on the clinical use of dentals stem cells or the use of dental stem cells as an intervention (Figure 7) [496,497,498,499,500,501,502]. The majority of the identified publications focus on dentistry; in detail, publications evaluated the role of dental stem cells in third molar post-extraction socket healing [496], the role of autologous periodontal ligament stem cells’ periodontal intrabony defects using [497], the role of dental stem cells in human intrabony defects [498], and the regeneration dental pulp after implantation into injured teeth [501], respectively. One publication on the clinical uses in dentistry was a case report reporting the grafting of mesenchymal stem cells from dental pulp for the retrieval of a periodontally compromised tooth by allogeneic [500].

**Figure 8:**
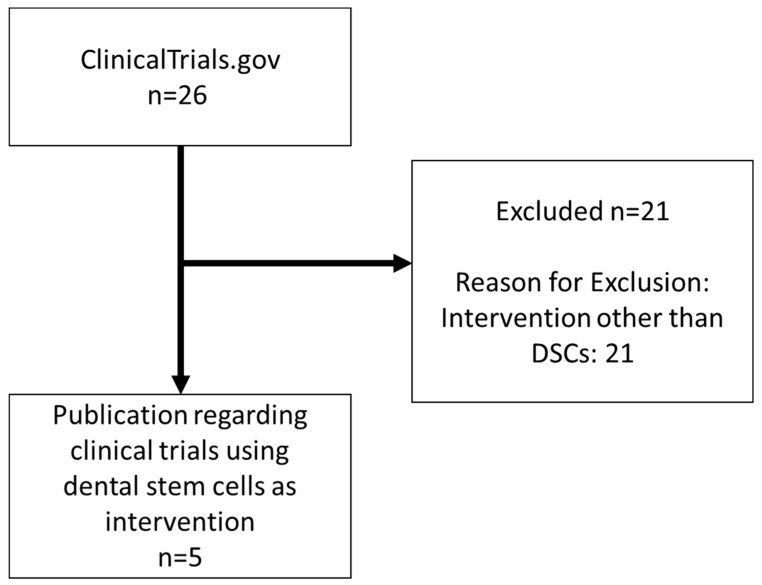
Systematic review flow chart of the systemic ClinicalTrials.gov database review. Out of the 26 currently active clinical trials, 21 trials had to be excluded, since they did not use dental stem cells for intervention, leaving a total of five clinical trials where DSCs were used as intervention.

A single study reports the planned use of DSCs in infections disease; in detail, it reports the clinical trial protocol for a clinical trial evaluating the use of allogeneic DPSCs to treat patients with severe COVID-19 [502]. The search of the clinical trial database clinicaltrials.gov identified 26 currently active clinical trials. Twenty-one trials had to be excluded, since they did not use dental stem cells for intervention (Figure 8). A total of five clinical trials were using DSCs as intervention in 204 patients (Table 3). All identified trials were industry-sponsored Phase I or Phase I/II trials. All trials were sponsored by companies headquartered in China. Two of the trials were investigating the role of DPSCs to treat COVID-19 [502] (ClinicalTrials.gov Identifier: NCT04336254, NCT04302519), one was investigating the role of DPSCs in a trial for osteoarthritis in the knee (NCT04130100), another was investigating the role of human dental pulp mesenchymal stem cells ischemic stroke (NCT04608838), and one was investigating the role of dental pulp cells for the treatment of diabetes (NCT03912480).

Even with the high number of publications about dental stem cells, a low number of publications clinically using dental stem cells were identified [496,497,498,499,500,501,502]. While the in vitro and animal in vivo experiments with DSCs seem promising, there is still plenty left to investigate whether it was concerning cellular behavior, culturing techniques, or choice of scaffolds approaches in order to better the translation of these techniques into clinical settings. Thus, it is not expected that the relative low number publication will change in the future, since only five clinical trials were registered in the clinicaltrials.gov database. It has also to be noted that all trials identified were Phase I or combined Phase I/II trials, which is an indication that all identified development programs are in an early development stage. However, the identified ongoing trials are not limed to hard tissue regeneration as the identified publications. The trials have a broader scope such as infections disease, orthopedics, cardiology, or diabetes.

## 10. Conclusions and Prospects

With this review, we aim to shed light on the potential of various types of dental stem cells, their use in basic research, and possible applications in regenerative medicine approaches. Dental stem cells are easy to obtain from medical waste, which is an advantage over most other adult stem cells sources. The cells can be young, e.g., if derived from tooth follicle and similar to umbilical cord-derived stem cells, which are also young, and the banking and cryopreservation of DSCs is possible. Due to their ecto-embryonal origin, DSCs are of major interest for approaches in hard tissue repair and regeneration. Thus, their osteogenic and angiogenic abilities were summarized herein. Moreover, details about the effects of growth factors and environmental cues that might improve their differentiations or enhance their functions are reported. However, more investigations are needed for optimized protocols, since so far, there are conflicting reports. Furthermore, DSCs can be immortalized to gain cell lines or be reprogrammed to iPSCs for long-term studies. DSCs-derived iPSCs were already used to gain cell types from dissenting lineages. In addition, several types of oral tissue organoids were generated, which can be used for disease modeling and subsequent pharmaceutical research. However, they are also used for developmental studies of dental tissues depending on the targeted stem cell type for organoid production. New therapy techniques using DSCs are discussed in this review, such as the formation of cell sheets or 3D constructs, but also cell-free approaches utilizing the DSCs secretome. Recent advances in scaffolds/biomaterials and the release of small molecules and growth factors from theses scaffolds were depicted as well. These data open the door for future applications in therapy such as for tissue regeneration, repair, and even organ replacement. The reported clinical trials with DSCs show great potential. However, there are little data so far. The quantity of clinical studies is quite small in comparison to the impressive amount of literature found on basic research. This clearly indicates the need for more efforts in translation. If this can be triggered by our review, we achieved our goal.

## Figures and Tables

**Table 1:** A Summary of the growth factors reported in this section and their effect on the osteogenic differentiation of dental stem cells.

Growth Factor(s)	Stem Cell Type	Effect on Osteogenesis	References
BMP2	DPSCs	Transfection and overexpression of BMP2 had no effect on osteogenesis	Tóth et al., 2020
	SHED	BMP2 increased osteogenesis in the presence or absence of osteogenic induction media	Casagrande et al., 2010 Billings et al., 2007 Koyama et al., 2009 Hara et al., 2011
	DFSCs	Activation of Wnt/β-catenin signaling with WNT3A suppresses the BMP2-mediated induction of osteoblasts of DFSCs and was also responsible for a downregulation of RUNX2, ALP, and OCN	Silvério et al., 2011
		Induction of osteogenesis in DFSCs by BMP2 and overexpression of DLX3 activated the NOTCH signaling pathway and increased osteogenesis	Viale-Bouroncle et al., 2014
	PDLSCs	BMP2 treatment was shown to improve osteogenesis	Hakki et al., 2013
		Transduction with BMP2 improved osteogenesis	Jung et al., 2014
BMP6	DFSCs	BMP6 supplementation restored the osteogenic differentiation of late-passage DFSCs	Yao et al., 2014
		BMP6 enhanced the gene expression level of RUNX2 and other osteogenic markers	Takahashi et al., 2013
	PDLSCs	BMP6 treatment was shown to improve osteogenesis. In comparison to BMP2 and BMP7 treatment, BMP6 treatment showed the highest mineralization	Hakki et al., 2013
BMP7	DPSCs	BMP7 increased osteogenesis in a dose-dependent matter	Zhu et al., 2018
	PDLSCs	BMP7 Improved the osteogenesis of PDLSCs	Açil et al., 2015
			Hakki et al., 2013
BMP9	DPSCs	Overexpression of BMP9 enhanced osteogenesis	Li et al., 2020
	DFSCs	BMP9 transfection increased the osteogenesis of DFSCs	Li et al., 2012
	PDLSCs	BMP9 transfection accompanied by electromagnetic pulses increased the osteogenesis	Wang et al., 2017
	PDLSCs	BMP9 improved osteogenesis through activation of the MAPK signaling pathway and the phosphorylation of p38 and ERK1/2	Ye et al., 2014
IGF1	DPSCs	IGF-1 promoted proliferation and osteogenic differentiation	Lv et al., 2016 and Feng et al., 2014
	PDLSCs	IGF1 improved proliferation and osteogenic capabilities of PDLSCs	Yu et al., 2012
VEGF	DPSCs	Stable overexpression of VEGF promoted osteo/odontogenic marker expression	Zhang et al., 2014
BFGF	SHED	BFGF treatment decreased osteogenesis	Osathanon et al., 2013 Nowwarote et al., 2015, 2018 Li et al., 2012
		Hypoxia and bFGF improved proliferation and osteogenic differentiation	Novais et al., 2019
	DPSCs	BFGF pretreatment for one week increased the osteogenic differentiation. Whereas 2 weeks pretreatment with bFGF decreased the in vitro osteogenic differentiation	Qian et al., 2015
		Addition of bFGF during the differentiation inhibited the osteogenesis	Del Angel-Mosqueda et al., 2015
		Treatment with bFGF increased osteo/odontogenesis in DPSCs; the effect increased with increasing concentrations of bFGF	Kim et al., 2010
FGF9	DPSCs	FGF9 decreased osteogenesis	Lu et al., 2015
TGF-β1	SHED	TGF-β1 increased osteogenesis of cells differentiated on a chitosan scaffold	Farea et al., 2014
EGF	DPSCs	Addition of bFGF during the differentiation enhanced the osteogenesis	Del Angel-Mosqueda et al., 2015
TGF-β2	DFSCs	Inhibition of TGF- β2 increased levels of TGF- β1, ALP activity, and Alizarin red S staining	Um et al., 2018
VEGF AND BMP2	DPSCs	Continuous treatment with VEGF enhanced the osteogenic differentiation Treatment with VEGF for 1 week and BMP2 throughout the differentiation enhanced osteogenesis to a lesser extent	Aksel and Huang, 2017
BFGF AND TGF-β1	DPSCs	BFGF promoted the proliferation of DPSCs, combination of bFGF and TGF-β1 enhanced osteogenesis	He et al., 2008
BFGF AND BMP2	PDLSCs	Consecutive treatment for 3 days bFGF followed with BMP2 for the duration of differentiation resulted in increased osteogenic differentiation	Kang et al., 2019
VEGF AND BFGF	PDLSCs	VEGF treatment promoted the mineralization and expression of osteogenic markers	Lee et al., 2012
IGF1 AND VEGF	CDPSCs	IGF-1 or VEGF alone promoted proliferation and osteogenesis, The combination of IGF-1 and VEGF further enhanced osteogenesis	Lu et al., 2019

**Table 2:** Materials applicable for loading, encapsulation, and drugs/signaling molecules release for promoting osteogenic and angiogenic stem cell differentiation, proliferation, and growth. Adapted and extended based on Baranova 2020 [484]. Copyright MDPI 2021.

Drugs (Loaded/Released)	Encapsulation/Release Material	Cells/Cell Lines and Target Tissue	Release Details and Results	Reference
FGF	Peptide hydrogels coated on hybrid scaffolds prepared from nanohydroxy-apatite/polyamide 66 (nHA/PA66)	Osteogenic differentiation of female SD rat BM-MSCs; in vivo test with induced large bone defects in female SD rats	Composite material offers stable sustained release of bFGF and improved osteogenesis in vitro and in vivo	Zhao et al., 2020]
	Acetyl chitosan (chitin) gel (for binding and release of chitin binding peptide-bFGF fusion protein)	Studies without using cell culture/biological assays	Lysozyme-responsive (dose-dependent or activity-dependent) release of CBP-FGF2	Tachibana et al., 2020
	Silk fibroin e-gel scaffolds (loaded with albumin = Fe_3_O_4_-βFGF conjugate)	SaOS-2 cells cultured on human serum albumin; osteogenic differentiation	Enhancing alkaline phosphatase, calcium deposition, collagen synthesis during osteogenic differentiation	Karahaliloğlu et al., 2017
BMP2	Porous silica-calcium phosphate composite (SCPC50)	Mongrel dog with induced mandible defect; osteogenic differentiation	Sustained release of rhBMP2 for alveolar ridge augmentation in saddle-type defect	Fahmy et al., 2015
	Calcium phosphate (Ca-P)/poly(L-lactic acid) (PLLA) nanocomposites	Human BM-MSCs; osteogenic differentiation	3D Ca-P-PLLA scaffold sustainably releasing Ca^2+^ and rhBMP2 for enhanced osteogenesis	Wang et al., 2017
	Poly(lactic-co-glycolic acid)-multistage vector composite microspheres (PLGA-MSV)	Male SD rat BM-MSCs; osteogenic differentiation	Controlled prolonged release of BMP2 for osteoinduction of rat BM-MSCs	Minardi et al., 2020
	VEGF (in fibrin gel) and BMP2 (in gelatin) used to coat dentine discs	Dental pulp stem cells (DPSCs); angiogenic and odontogenic differentiation	Both systems allowed the controlled releases of growth factors	Aksel et al., 2018
TGF-β	Poly(ethylene oxide terephthalate)/poly(butylene terephthalate) (PEOT/PBT) fibrous resins	TK173 (human renal fibroblast cell line), neonatal rat dermal fibroblasts (nRDFs)	Sustained delivery of growth factors (TGF-β1, PDGF-ββ, IGF-1) using a layer-by-layer assembly for supporting fibroblast attachment and proliferation	Damanik et al., 2020
	Poly(vinylidene fluoride) (PVDF) nanofibers fabricated via electrospinning method with/without chitosan nanoparticles	Adipose tissue-derived mesenchymal stem cells (A-MSCs); smooth muscle cell (SMC) differentiation	PVDF-TGF-β1 as a biofunctional scaffold and release material for enhancing SMC differentiation	Ardeshirylajimi et al., 2018
	Alginate nanogel with cross-junction microchannels	Human MSCs, chondrogenic differentiation	Controlled release of TGF-β3 from polymeric nanogel for enhanced chondrogenesis	Mahmoudi et al., 2020
	Chitosan-based 3D interpenetrating pore structures of large internal surface area	Primary human periodontal ligament stem cells (PDLSCs); osteogenic differentiation	Controlled release of TGF-β3 from chitosan sponge for the repair of periodontal soft and hard tissue defects	Li et al., 2019
	GelMA hydrogel columns in situ fixed in hollowed channels of 2-N,6-O-sulfated chitosan (26SCS) for dual release	Human umbilical vein endothelial cells (HUVECs)-Red Fluorescent Protein (HUVECs-RFP) and human bone marrow stromal cells (BM-MSCs); osteogenic and angiogenic differentiation	Dual release of VEGF and rhBMP2 including release kinetics. Improved osteogenic and angiogenic differentiation (in vitro and in vivo)	Tang et al., 2019
ATP, suramin (P2XR activators)	Albumin nanoparticles (aNPs) of low polydispersity loaded with ATP and coated with erythrocyte membrane (EM)	HeLa, HEK-293 cell lines	EM-aNPs developed as a delivery vehicle for ATP to be used as an anticancer agent	Díaz-Saldívar et al., 2019
	Hydroxyapatite (HA)/agarose hybrids for ATP and suramin release	Release kinetic studies w/o cells; biocompatibility test using A-MSCs and MG-63 cell line	ATP and suramin release for hard tissue formation	Witzler et al., 2019b
Purmorphamine (Hh activator/Smo agonist)	Glutaraldehyde (GA)–cross-linked gelatin type B matrix (for small molecules and proteins release)	Release kinetics (burst vs. sustained release) studied without using cell culture; released molecules bioactivity verified in cell culture/biological assays	In vitro delivery system for Wnt, Hh agonists, and growth factors (e.g., FGF2, VEGF) beneficial for endochondral ossification	Ahrens et al., 2017
	Poly(propylene glycol-co-lactide) dimethacrylate (PPLM) adhesives for incorporating purmorphamine and TCP	MC3T3-E1 (mouse pre-osteoblast cell line); proliferation of pre-osteoblast cells (MC3T3-E1)	Cell attachment and response to photocured, degradable bone adhesives containing TCP and purmorphamine	Gellynck et al., 2011
	Poly(caprolactone) (PCL) microspheres for encapsulating small molecules using a single emulsion oil-in-water method	Human-induced pluripotent stem cell (iPSC) aggregates differentiating into motor neurons	Prolonged release during neural differentiation	De la Vega et al., 2018
BIO (Wnt/β-catenin activator)	Polymersomes (PMs) consisting of poly(ethylene glycol) PEG-PCL block copolymer (approved for clinical use)	Murine 3T3 Wnt reporter cells and human BM-MSCs; osteogenic differentiation	Controlled activation of Wnt signaling and Runx2 during osteogenesis	Scarpa et al., 2018

**Table 3:** Clinical trials using dental stem cells as intervention.

NCT Number	Title	Conditions	Interventions	Study Designs	Phases	Sponsor	Enrollment	Funding Source
NCT04336254	Safety and Efficacy Study of Allogeneic Human Dental Pulp Mesenchymal Stem Cells to Treat Severe COVID-19 Patients	COVID-19	Allogeneic DPSCs	Randomized Parallel Assignment Triple Blind Study	Phase 1/Phase 2	Beijing SH Bio-Tech Corporation, Beijing (CN)|Utooth Biological Technology Co., Ltd. Hubei (CN)	20	Industry
NCT04302519	Novel Coronavirus Induced Severe Pneumonia Treated by Dental Pulp Mesenchymal Stem Cells	COVID-19	DPSCs	Single Arm; Open Label	Early Phase 1	CAR-T (Shanghai) Biotechnology Co., Ltd.	24	Industry
NCT04130100	Clinical Study of Pulp Mesenchymal Stem Cells in the Treatment of Primary Mild to Moderate Knee Osteoarthritis	Knee Osteoarthritis	DPSCs	Randomized; Parallel Assignment; Open Label	Early Phase 1	CAR-T (Shanghai) Biotechnology Co., Ltd.	60	Industry
NCT04608838	A Randomized Placebo-Controlled Multicenter Trial to Evaluate the Efficacy and Safety of JTR-161, Allogenic Human Dental Pulp Stem Cell, in Patients with acute Ischemic stroke (J-REPAIR)	Acute Ischemic Stroke	Allogenic DPSCs	Randomized; Parallel Assignment, Quadruple Blind	Phase 1/Phase 2	Teijin Pharma Limited	76	Industry
NCT03912480	Stem Cells from Human Exfoliated Teeth in Treatment of Diabetic Patients with Significantly Reduced Islet Function	Type 1 Diabetes	DPSCs	Single Group Assignment; Open Label	Early Phase 1	CAR-T (Shanghai) Biotechnology Co., Ltd.	24	Industry

## Data Availability

Not applicable.

## Abbreviations

ASCs	Adult stem cells
A-MSCs	Adipose tissue-derived mesenchymal stem cells
aNPs	Albumin nanoparticles
ALP	Alkaline phosphatase
BM-MSCs	Bone marrow mesenchymal stem cells
BMP	Bone morphogenetic protein
BSP	Bone sialoprotein
CDPSCs	Carious dental pulp stem cells
EpiSC	Dental epithelial stem cells
DFCs	Dental follicle cells
DFSCs	Dental follicle stem cells
DPSCs	Dental pulp stem cells
SCCD	Dental pulp stem cells from carious deciduous teeth
DSCs	Dental stem cells
DMP1	Dentin matrix acidic phosphoprotein 1
DPCs	Dental pulp cells
DSPP	Dentin sialophosphoprotein
DLX3	Distal-less homeobox 3
DLX4	Distal-less homeobox 4
ESCs	Embryonic stem cells
ECs	Endothelial cells
EM	Erythrocyte membrane
ERK1/2	Extracellular signal-related kinase 1/2
FGF	Fibroblast growth factor
G-MSCs	Gingival mesenchymal stem cells
HGF	Hepatocyte growth factor
HERS/ERM	Hertwig’s epithelial root sheath/epithelial rests of Malassez
hTERT	Human telomerase reverse transcriptase
HUVECs	Human umbilical vein endothelial cells
HIFs	Hypoxia-inducible factors
iMSCs	Induced pluripotent stem cells derived mesenchymal stem cells
iPSCs	Induced pluripotent stem cells
IGF-1	Insulin-like growth factor-1
Klf4	Kruppel like factor 4
LLLT/I	Low-level laser therapy/ irradiation
MEPE	Matrix extracellular phosphoglycoprotein
MSCs	Mesenchymal stem cells
mtDNA	Mitochondrial DNA
MAPK	Mitogen-activated protein kinase
Oct3/4	Octamer-binding transcription factors 3/4
OCN	Osteocalcin
OPN	Osteopontin
OPG	Osteoprotegerin
OSX	Osterix
PAX9	Paired box gene 9
PCy-MSCs	Periapical cyst-mesenchymal stem cells
PDLSCs	Periodontal ligament stem cells
PI3K	Phosphoinositide 3-kinase
PDGFs	Platelet-derived growth factors
PSCs	Pluripotent stem cells
PDLSCs	Primary periodontal ligament stem cells
rhBMP7	Recombinant human bone morphogenic protein 7
RUNX2	Runt-related transcription factor 2
SMC	Smooth muscle cells
Sox2	SRY (sex determining region Y)-box 2
SSEA	Stage-specific embryonic antigens
SHED	Stem cells from exfoliated deciduous teeth
SCAP	Stem cells from the apical papilla
SDF-1α	Stromal-derived factor-1α
TGSCs	Tooth germ stem cells
TGF-β	Transforming group factors-beta
UC-MSCs	Umbilical cord mesenchymal stem cells
VEGF	Vascular endothelial growth factor
WJ-MSCs	Wharton’s Jelly mesenchymal stem cells
